# A Pro‐Regenerative Supramolecular Prodrug Protects Against and Repairs Colon Damage in Experimental Colitis

**DOI:** 10.1002/advs.202304716

**Published:** 2024-01-21

**Authors:** Kelsey G. DeFrates, Elaine Tong, Jing Cheng, Ellen Heber‐Katz, Phillip B. Messersmith

**Affiliations:** ^1^ Department of Bioengineering University of California, Berkeley Berkeley CA 94720 USA; ^2^ Lankenau Institute for Medical Research Wynnewood PA 19096 USA; ^3^ Department of Materials Science and Engineering University of California, Berkeley Berkeley CA 94720 USA; ^4^ Materials Sciences Division Lawrence Berkeley National Laboratory Berkeley CA 94720 USA

**Keywords:** HIF‐1α, inflammatory bowel disease, prolyl hydroxylase inhibitor, regeneration

## Abstract

Structural repair of the intestinal epithelium is strongly correlated with disease remission in inflammatory bowel disease (IBD); however, ulcer healing is not addressed by existing therapies. To address this need, this study reports the use of a small molecule prolyl hydroxylase (PHD) inhibitor (DPCA) to upregulate hypoxia‐inducible factor one‐alpha (HIF‐1α) and induce mammalian regeneration. Sustained delivery of DPCA is achieved through subcutaneous injections of a supramolecular hydrogel, formed through the self‐assembly of PEG‐DPCA conjugates. Pre‐treatment of mice with PEG‐DPCA is shown to protect mice from epithelial erosion and symptoms of dextran sodium sulfate (DSS)‐induced colitis. Surprisingly, a single subcutaneous dose of PEG‐DPCA, administered after disease onset, leads to accelerated weight gain and complete restoration of healthy tissue architecture in colitic mice. Rapid DPCA‐induced restoration of the intestinal barrier is likely orchestrated by increased expression of HIF‐1α and associated targets leading to an epithelial‐to‐mesenchymal transition. Further investigation of DPCA as a potential adjunctive or stand‐alone restorative treatment to combat active IBD is warranted.

## Graphical abstract

In Inflammatory Bowel Disease, structural repair of the intestinal epithelium is essential to achieving clinical remission. This article shows that non‐local delivery of a small molecule prolyl hydroxylase inhibitor can preserve and restore barrier function in a mouse model of experimental colitis. These effects are orchestrated by stabilization of the transcription factor, hypoxia inducible factor one‐alpha in the colon.

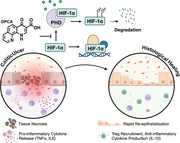

## 1 Introduction

IBD is a chronic condition of unknown etiology, characterized by inflammation and ulceration of the colon and rectum. For patients with IBD sub‐disorders ulcerative colitis (UC) or Crohn's Disease (CD), relapsing and remitting periods of abdominal pain, diarrhea, and rectal bleeding result in physical discomfort and greatly impact the psychological, social, and professional aspects of life.^[^ ^1^ ^]^ Although recent progress in the use of corticosteroids and novel biologics has served to mitigate these symptoms and IBD‐related fatalities, mucosal restitution and histological healing are rarely achieved,^[^ ^2^, ^3^ ^]^ leaving weakened bowel tissue that is incapable of blocking the diffusion of antigens into the lamina propria to restore immunological homeostasis. As a result, the majority of patients with UC and CD never achieve disease remission.^[^ ^1^, ^4^ ^]^ In severe forms of CD, where tissue damage extends beyond the epithelium, transmural lesions are also highly susceptible to fibrosis, leading to the formation of penetrates and strictures that must be corrected by surgical intervention.^[^ ^5^ ^]^ Similar fibrostenotic complications have also been reported in 8% of UC cases.^[^ ^6^ ^]^ With incidence of IBD growing throughout the Western world and newly industrialized regions, there is significant clinical need for new therapeutics that not only attenuate inflammation but catalyze the regeneration of damaged bowel tissue to prevent disease relapse and treatment escalation.

Recently, short‐term treatment with 1,4‐dihydrophenathrolin‐4‐one‐3‐carboxylic acid (DPCA), a small molecule PHD inhibitor, has been shown to result in regeneration in adult mammals.^[^ ^7^, ^8^, ^9^, ^10^ ^]^ These regenerative effects are thought to be driven by DPCA‐induced stabilization of HIF‐1α.^[^ ^7^, ^8^, ^9^, ^10^ ^]^ Although constitutively expressed in all cells, HIF‐1α is usually rapidly degraded in normoxia through the action of PHD enzymes, which mark the factor for ubiquitin‐dependent proteasomal degradation.^[^ ^11^, ^12^ ^]^ In the presence of DPCA, PHD activity is inhibited, allowing HIF‐1α to accumulate and translocate to the nucleus, where it initiates the expression of genes associated with cell survival, migration, and metabolism.^[^ ^7^, ^8^, ^9^, ^10^ ^]^ In several naturally regenerative species such as zebrafish and the Murphy Roths Large (MRL) laboratory mouse strain, augmented HIF‐1α expression after injury has been shown to play a direct role in healing by influencing progenitor cell plasticity, inflammation, and tissue remodeling.^[^ ^13^, ^14^, ^15^, ^16^, ^17^, ^18^ ^]^

Pharmacological enhancement of HIF‐1α expression in non‐healing mammals through short‐term delivery of DPCA represents a clinically scalable, cell‐free alternative to conventional approaches in regenerative medicine. Previously, we described a polymeric prodrug delivery system for DPCA, consisting of DPCA coupled to a trivalent end‐functionalized poly(ethylene) glycol (PEG) via a hydrolyzable ester linkage.^[^ ^8^ ^]^ In water, the amphiphilic nature of PEG‐DPCA drove self‐assembly to form supramolecular nanofibers, measuring several microns in length.^[^ ^8^, ^19^ ^]^ Physical entanglement of nanofibers, aided by cross‐linking with a telechelic PEG‐DPCA, resulted in the formation of a shear‐thinning gel suitable for subcutaneous injection and timed release of DPCA. Using this strategy, full‐thickness ear hole wounds in non‐regenerating mice showed complete closure and tissue regeneration, whereas wounds in untreated mice remained unhealed.^[^ ^8^ ^]^ While ear hole wound model allowed us to validate the performance of our drug delivery vehicle, it did not represent a clinically relevant injury or disease. Given the ubiquitous expression of HIF‐1α across organ systems, we hypothesize that DPCA may be able to regenerate many types of injuries, even those associated with host‐mediated damage and inflammation, such as IBD ulcers.

Therefore, the aim of this study was to evaluate the capacity of DPCA to promote histological healing in a murine model of experimental colitis to fulfill the unmet clinical need for pro‐regenerative therapies in IBD care. Using the PEG‐DPCA hydrogel previously developed by our laboratory, we show that two nonlocal injections of the gel prevent disease onset in a DSS‐induced colitis model.^[^ ^8^ ^]^ When administered after disease onset, one injection of the gel was also found to accelerate re‐epithelialization and weight gain. We found that this response was accompanied by increased expression of pro‐regenerative cytokines and downstream targets of HIF‐1α signaling known to regulate epithelial cell migration and intestinal barrier properties. Our results suggest that short‐term administration of DPCA may be a restorative therapy for IBD.

## 2 Results

### 2.1 Short‐Term Treatment with DPCA Accelerates Weight Recovery and Ulcer Healing in DSS‐Induced Colitis

In IBD, changes to the microvascular network within the bowels combined with excessive inflammation, have been shown to contribute to the development of a hypoxic colon.^[^ ^20^, ^21^ ^]^ In this state, prophylactic activation of HIF‐1α has been shown to lead to the induction of barrier‐protective genes such as intestinal trefoil factor 3 *(TFF3)* and p‐glycoprotein (*P‐GP)*, which serve to mitigate mucosal damage in active UC.^[^ ^22^ ^]^ While upregulation of HIF‐1α in myeloid cells is thought to exacerbate inflammation, stabilization of the protein in colonic epithelial cells was accompanied by a reduction in levels of pro‐inflammatory cytokines, tumor necrosis factor alpha (TNF‐α) and interferon gamma (IFN‐γ).^[^ ^23^ ^]^ Recently, studies have shown that loss of HIF‐1α in murine models of colitis worsens disease symptoms, while increased levels of the protein were protective in disease progression.^[^ ^22^ ^]^ These findings, combined with our past work on HIF‐1α‐induced regeneration, led us to hypothesize that pre‐treatment with DPCA may confer protection against ulcer formation and disease progression in experimental colitis.

To investigate this hypothesis, we utilized a murine model of DSS‐induced acute colitis, established by spiking cage drinking water with 1.5% DSS for 10 days. This treatment is known to result in epithelial erosion throughout the colon, which facilitates the movement of luminal antigens into the mucosa and submucosal space. This then catalyzes a pro‐inflammatory cascade that perpetuates tissue damage. In response, mice exhibit pronounced weight loss, changes in stool consistency, and hematochezia.^[^ ^24^, ^25^ ^]^ Characteristic changes in bowel architecture such as goblet cell depletion, epithelial erosion, submucosal thickening, and cryptitis closely resemble features of human IBD, making the DSS‐induced colitis model well‐suited for studying the effects of DPCA on ulcer repair.^[^ ^24^ ^]^

**Figure 1:**
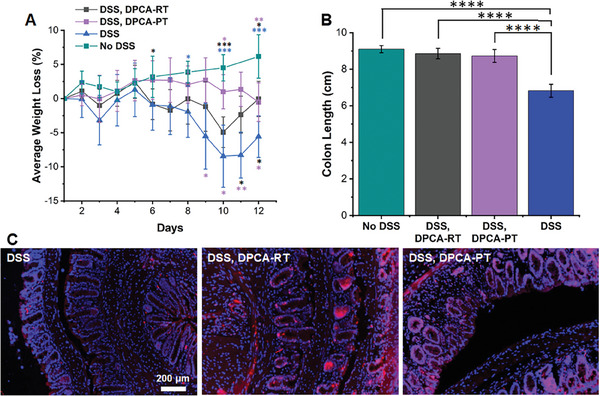
A) Average change in weight for mice exposed to 1.5% DSS in drinking water over 10 days or normal drinking water (No DSS, green). Preventative treatment groups (DPCA‐PT) received injections of DPCA prodrug on day −2 and day 5, whereas restorative treatment groups (DPCA‐RT) received one injection after disease onset on day 5. Relative to untreated controls (DSS), the DPCA‐PT treatment group showed significantly less weight loss. Weight recovery in the DPCA‐RT treatment group was also accelerated following the removal of DSS on day 10. However, both groups showed more weight loss relative to healthy controls, although this was less than untreated mice. B) At sacrifice (day 12), colons from DPCA‐treated mice retained healthy length, despite exposure to DSS, while untreated, DSS‐only controls, exhibited significant shrinkage. C) These effects are likely driven by upregulation of DPCA's main therapeutic target, HIF‐1α in the colon (red, DAPI counterstain). (Student's *t*‐test, *N* = 5, ^*^ *p* < 0.05; ^**^ *p* < 0.01, ^***^ *p* < 0.001, ^****^ *p* < 0.0001. In A, black^*^ corresponds to significance between DSS, DPCA‐RT, and DSS (bottom), or No DSS (top); purple^*^ corresponds to significance between DSS, DPCA‐PT, and DSS (bottom), or No DSS (top), and blue^*^ corresponds to significance between DSS and No DSS).

To deliver DPCA, we utilized a shear‐thinning hydrogel, formed from the self‐assembly of PEG‐DPCA conjugates (see SI methods and Figure S1, Supporting Information).^[^ ^8^ ^]^ When administered subcutaneously in the back of the neck in adult mice, gradual release of DPCA was found to stabilize HIF‐1α in‐ear hole wounds and induce complete regeneration of both hard and soft tissues of the periodontium.^[^ ^8^, ^9^, ^10^ ^]^ Pursuing a similar treatment regimen, in this study we administered doses of PEG‐DPCA gel in the DSS‐induced colitis model on day −2 (before DSS exposure) and day 5 (during DSS exposure). From days 0 to 10, cage water was spiked with 1.5% DSS, and animal weight was monitored daily. On day 10, DSS was replaced with normal drinking water and animals were given two days to recover before sacrifice. As seen in **Figure** **1**A, untreated mice exposed to 1.5% DSS exhibited rapid weight loss and pronounced colon shortening (Figure 1B). In the DPCA pre‐treated group, however, mice maintained their original body weight despite challenge with DSS. Although overall weight gain was less than healthy mice, they exhibited significantly less weight loss compared to DSS only controls. At sacrifice on day 12, mice in the DPCA‐PT group also had significantly longer colons, comparable to healthy controls (Figure 1A,B).

Since pretreatment with DPCA appeared to mitigate the symptoms of DSS‐induced colitis, we wondered if delivery after the onset of disease would accelerate recovery. Therefore, to test DPCA as a restorative treatment, we administered one injection of the DPCA gel at day 5 (DPCA‐RT) of the 1.5% DSS regimen. On day 10, we again removed DSS from the drinking water and monitored animal recovery for the next two days. During this time, DPCA‐RT mice exhibited significantly more weight gain, while untreated colitic controls showed only minor improvements (Figure 1A). In both groups, healthy weight was not restored, likely due to the short recovery time. Akin to mice in the preventative treatment group, those that received a single injection of DPCA also presented with normal colon length at day 12 (Figure 1B). Remarkably, these effects were observed after delivery of only 5 µg/g body weight of drug, a level that is over 20x lower than previous reports on the use of PHD inhibitors for IBD.^[^ ^23^ ^]^

It is important to note that the prodrug hydrogel was administered non‐locally, i.e., subcutaneously in the upper back of the mouse. Nevertheless, we observed upregulation of HIF‐1α in the colon (Figure 1C; Figure S2, Supporting Information). We quantified fluorescence staining of HIF‐1α across tissues from mice in all treatment groups and determined that treatment with DPCA resulted in a 1.3x higher average integrated density value for both DPCA‐RT and DPCA‐PT (Figure S3, Supporting Information). Our observations of HIF‐1α stabilization distant from the prodrug administration site is consistent with our previous reports showing non‐local injections of DPCA hydrogels stabilize HIF‐1α in ear and periodontal tissues.^[^ ^7^, ^8^, ^9^, ^10^ ^]^ In agreement with our previous studies, we also did not observe a significant change in HIF‐2α expression in the colon (Figure S4, Supporting Information).^[^ ^7^ ^]^ In fact, untreated colitic mice appeared to exhibit higher expression of HIF‐2α compared to DPCA‐treated mice, likely due to local tissue hypoxia perpetuated by inflammation and vascular damage.^[^ ^26^ ^]^ HIF‐1α selectivity is especially relevant in IBD care, since upregulation of HIF‐2α in the colon has been shown to drive inflammation and tumorigenesis.^[^ ^27^ ^]^ Thus, this finding also distinguishes our approach from previous attempts to modulate HIF in IBD using the PHD inhibitor FG‐4497, which is known to upregulate both HIF‐1α and HIF‐2α.^[^ ^23^, ^28^ ^]^

**Figure 2:**
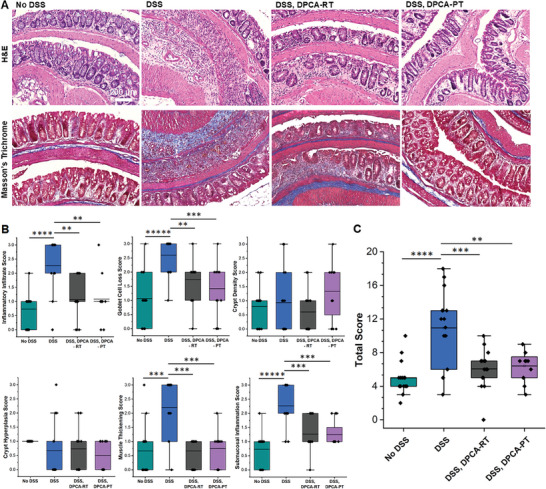
After exposure to DSS, untreated mice appeared to exhibit pronounced inflammation and epithelium erosion throughout the colon. A) In contrast, DPCA‐treated mice appeared to retain normal tissue architecture with minimal infiltration and swelling in the submucosal space, minimal loss of goblet cells, and little evidence of increased collagen deposition. B) Blind grading of H&E sections corroborates histological healing or preservation in DPCA‐PT/RT mice compared to DSS controls. C) Overall, mice receiving DPCA before or after the onset of DSS‐induced colitis scored lower for markers of disease. See Experimental Section Table 1 for details on scoring. (Student's *t*‐test, *N* = 5 (DSS and DSS, DPCA‐RT) or 4 (DSS, DPCA‐PT) with 12 images reviewed per animal, ^*^ *p* < 0.05; ^**^ *p* < 0.01, ^***^ *p* < 0.001, ^****^ *p* < 0.0001, ^*****^ *p* < 0.00001).

We investigated whether improvements in weight maintenance and recovery following preventative and restorative treatment with DPCA were due to histological healing of inflammatory ulcers. Hematoxylin and eosin (H&E) staining of colon tissue samples verified that untreated colitic mice presented with inflammatory infiltrates, goblet cell loss, muscle thickening, and submucosa inflammation. In contrast, bowel tissue of DPCA‐treated mice was nearly analogous to healthy controls (**Figure** **2**A), with animals in both DPCA treatment groups showing only minor signs of submucosal inflammation (Figure 2B). Although a slight change in goblet cell numbers were noted in the restorative group compared to healthy tissue, it was not as severe as DSS only controls. Overall, colon tissue from DPCA‐treated mice showed less histological markers of disease compared to untreated controls and did not significantly differ from tissue taken from healthy animals, when judged using an established rubric for scoring of disease severity (see **Table** **1**) (Figure 2C).^[^ ^29^ ^]^ We also found no evidence of scar tissue formation in DPCA‐treated mice as assessed by Mason's trichrome staining for collagen, consistent with the rapid recovery of histological features and tissue regeneration (Figure 2A).

**Table 1:** Grading rubric for scoring of H&E images.

Score	Inflammatory Infiltrate	Goblet Cell Loss	Crypt Density Reduction	Crypt Hyperplasia	Muscle Thickening	Submucosal Inflammation
0	None	None	Normal	None	None	None
1	Increased presence of inflammatory cells	< 10%	< 10%	Slight increase in length	slight	individual cells
2	Infiltrates also in submucosa	10–50%	10 – 50%	2–3 fold increase in length	strong	infiltrate(s)
3	Transmural	> 50%	> 50%	> 3‐fold increase	excessive	large infiltrate(s)

### 2.2 Treatment with DPCA Attenuates Inflammation and Upregulates Factors Associated with Barrier Performance

**Figure 3:**
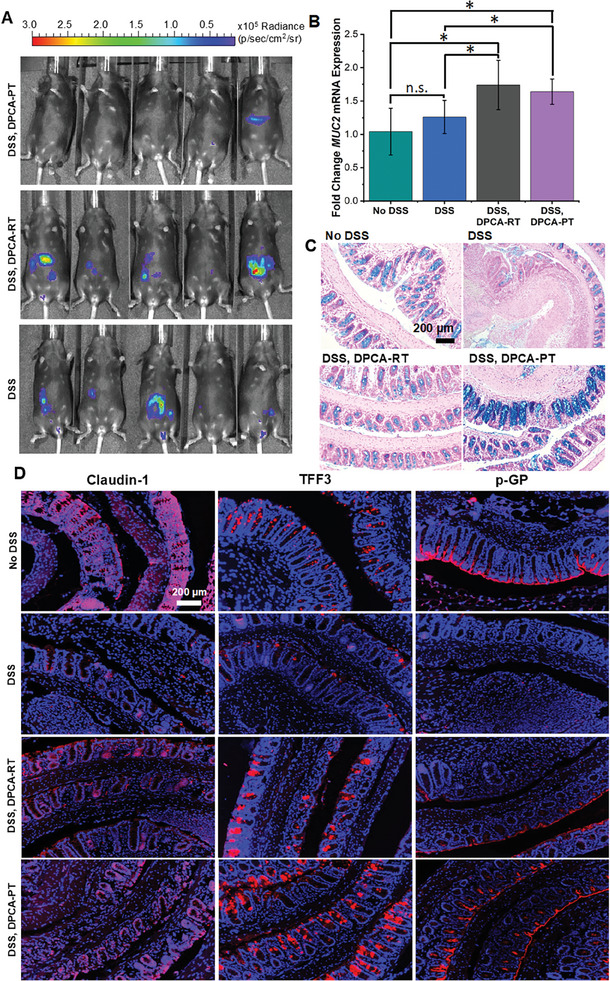
Inflammation in all mice was assessed using a chemiluminescent ROS‐responsive probe on day 9. DPCA‐PT mice that received DSS over 9 days showed little to no ROS activity. A) In contrast, untreated colitic controls (DSS) and mice in the restorative treatment group (DSS, DPCA‐RT), exhibited high signal throughout their lower abdomen, suggesting inflammation throughout the lower GIT. B) DPCA‐treated mice from both groups had increased expression of MUC2, C) as well as Alcian Blue staining for acidic mucins, which may strengthen barrier defense against DSS. D) HIF‐1α targets known to increase intestinal barrier strength, including Claudin‐1, TFF3, and P‐GP, are also highly expressed in DPCA treatment groups, comparable to healthy controls, unlike untreated colitic controls (red, DAPI counterstain). (Student's *t*‐test *N* = 3 (No DSS), 4 (DSS, DPCA‐PT) or 5 (DSS and DSS, DPCA‐RT) ^*^ *p* < 0.05).

In addition to assessing disease severity through weight loss and tissue histology, we evaluated inflammation in untreated and DPCA‐treated mice. Using the chemiluminescent reactive oxygen species (ROS) reporter L‐012, we verified that mice pretreated with DPCA did not develop inflammatory colitis. As seen in **Figure** **3**A, little evidence of ROS activity could be detected in four out of five DPCA‐PT treated mouse colons despite 9 days of exposure to DSS (Figure S5, Supporting Information). In contrast, untreated colitic mice (DSS) exhibited high luminescence localized to the GIT and extending down to the rectum at this same time point, although normalized luminescence between the groups was determined to be not statistically relevant (p > 0.05) (Figure S5, Supporting Information).

Given the role of HIF‐1α in intestinal maintenance, we hypothesized that pre‐treatment with DPCA may strengthen GIT barrier function and prevent DSS‐induced necrosis.^[^ ^30^ ^]^ In the colon, the epithelium is protected by a mucosal layer composed primarily of composed primarily of mucin 2 (MUC2), which is regulated by HIF‐1α.^[^ ^31^ ^]^ In the DSS model of induced colitis, loss of MUC2 is accompanied by more severe histological damage.^[^ ^32^ ^]^ In agreement with our hypothesis, we found that mice treated with DPCA exhibited increased expression of mRNA for MUC2, as well as greater staining for acidic mucins (Figure 3B,C). HIF‐1α targets known to be involved in barrier maintenance, such as tight junction protein Claudin‐1, TFF3, and P‐GP were also upregulated in DSS, DPCA‐PT treated mice compared colitic controls (Figure 3D). This effect was also seen in treatments groups receiving only one restorative dose of DPCA (DSS, DPCA‐RT) (Figure 3D). In this instance, however, barrier strengthening did not appear to mitigate inflammation at day 9, since DPCA‐RT mice exhibited ROS activity analogous to untreated colitic controls (Figure 3A). This suggests that DPCA accelerated histological healing of the damaged epithelium, since little to no ulceration was observed by day 12. In contrast to the pretreatment group, as well as previous work on PHD inhibitors in IBD care, this restorative treatment regimen (DSS, DPCA‐RT), better recapitulates clinical IBD therapies that are used to treat active disease.^[^ ^23^ ^]^

**Figure 4:**
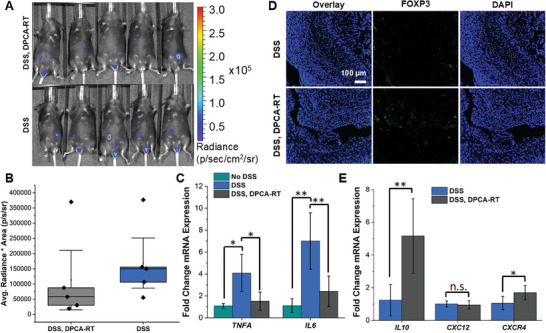
A) By day 12, ROS expression in the lower abdomen is reduced in some mice treated with DPCA‐RT. B) Chemiluminescent signal was normalized to area for all mice. Although the difference between average radiance in DSS and DSS, DPCA‐RT groups is not statistically different, four out of five mice that received DPCA showed lower levels of inflammation. C) At day 12, anti‐inflammatory signaling also appeared to be upregulated in DSS, DPCA‐RT mice compared to untreated colitic controls, which showed a drop in mRNA expression of IBD markers *IL6* and *TNFA*. D) Increased staining of Treg cell marker FOXP3 was also seen in DSS, DPCA‐RT treated mice, but not in colitic controls. E) mRNA for *IL10*, an anti‐inflammatory cytokine produced by Tregs was also highly expressed in drug‐treated mice, as well as *CXCR4*, which is essential for immune cell homing. (Student's *t*‐test, *N* = 4 For DSS, DPCA‐RT IL10, *N* = 5 (DSS and DSS, DPCA‐RT) or *N* = 3 (No DSS); n.s. = not significant; ^*^ *p* < 0.05; ^**^ *p* < 0.01).

To validate the restorative power of DPCA in IBD care, we again assessed ROS activity at day 12 in the DSS, DPCA‐RT treatment group. Although average radiance did not show a statistically significant difference compared to untreated controls (DSS), four out of five mice showed reduced chemiluminescence signal after treatment with DPCA (**Figure** **4**A,B). Although mice originally exhibited signs of inflammation at day 9, by day 12 the DSS, DPCA‐RT treatment group also showed lower mRNA expression for genes encoding for pro‐inflammatory cytokines, interleukin‐6 (*IL6*), and tumor necrosis factor alpha (*TNFA*), compared to untreated colitic controls (Figure 4B). In murine macrophage cultures, 24‐hour treatment with DPCA also appeared to attenuate TNFα production at both the mRNA and protein level, even after endotoxin challenge with liposaccharide (LPS) (Figure S6, Supporting Information). These findings are consistent with previous work showing that DPCA was capable of attenuating inflammation in a murine model of periodontitis.^[^ ^9^ ^]^ In this study, increased homing of T regulatory cells (Treg) to the site of injury may be facilitating this response. In colitic mice treated with one injection of DPCA, we also noted increased staining of Treg marker forkhead box P3 (FOXP3) throughout the submucosal space (Figure 4C) and increased mRNA expression for interleukin‐10 (*IL10*), an anti‐inflammatory cytokine produced by Tregs (Figure 4D). We suspect that DPCA‐induced stabilization of HIF‐1α may impact Treg cell homing through the C‐X‐C motif chemokine receptor 4 (CXCR4) pathway.^[^ ^33^ ^]^ While we did not find a significant increase in mRNA expression of the CXCR4 ligand CXCL12, mRNA expression for *CXCR4* was elevated in DPCA‐treated mice (Figure 4E). Recently Foxp3^+^ Tregs have been shown to aid in epithelial repair in experimental colitis, and Treg cell suppression or dysfunction is known to accelerate lesion formation in clinical IBD.^[^ ^34^, ^35^, ^36^ ^]^ Thus, HIF‐1α‐orchestrated recruitment of Tregs to active ulcers may directly contribute to healing in DPCA‐treated mice.

### 2.3 DPCA Repairs the Colon Lining through Activation of Epithelial Cells

**Figure 5:**
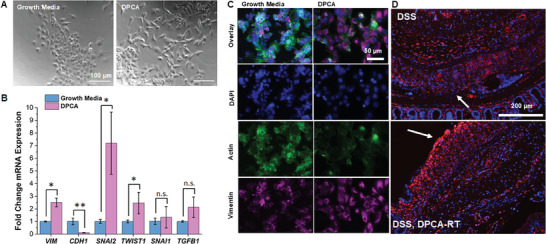
A) After 48‐hour treatment with DPCA, epithelial cells derived from murine colons adopt an elongated phenotype that is more closely associated with cells of the mesenchymal lineage. mRNA expression of mesenchymal marker, vimentin (*VIM*) is also upregulated, while epithelial marker, E‐cadherin (*CDH1*), is downregulated. B) Transcription factors associated with EMT also show increased expression at the mRNA level. C) Vimentin expression at the protein level is verified (magenta) and actin depolymerization (green) is also observed in DPCA‐treated samples which is usually associated with higher cell mobility. D) In regenerating epithelium, DSS, DPCA‐RT mice show higher intensity staining for vimentin compared to wounded epithelium in DSS only controls (white arrows). (DAPI counterstain). (Student's *t*‐test, *N* = 2 (Growth media) or 3 (DPCA), n.s. = not significant; ^*^ *p* < 0.05; ^**^ *p* < 0.01).

At the conclusion of our study, DSS, DPCA‐RT mice showed considerable improvements in the colon lining, despite initial signs of disease (weight loss and inflammation). This suggests that treatment with DPCA accelerates epithelial repair. Previously, DPCA was shown to promote a similar effect in a murine ear hole wound, where re‐epithelialization is seen just two days after injury.^[^ ^7^ ^]^ To better understand the mechanism driving this effect, we treated primary epithelial cells from mouse colons with DPCA. We found that after 48 h, drug‐treated colonic epithelial cells lost their characteristic “cuboidal” morphology and instead adopted an elongated, spindle‐like appearance (**Figure** **5**A). This phenotypic change is indicative of an epithelial‐mesenchymal transition (EMT), which is known to be a downstream effect of HIF‐1α signaling.^[^ ^37^, ^38^ ^]^ In support of an EMT, we also noted increased expression of the mesenchymal marker, vimentin, and significant actin depolymerization compared to untreated cells (Figure 5B,C). Increased mRNA expression of mesenchymal marker, vimentin (*VIM*), and loss of epithelial markers such as E‐cadherin (*CDH1*), were also observed in DPCA‐treated cells (Figure 5B). While vimentin was universally expressed throughout the lamina propria and submucosal space in both treated and untreated mice, expression was drastically reduced at the margins of colitic ulcers in untreated controls (Figure 5D). The regenerating epithelium around rare lesions in DPCA‐treated colitic mice, however, showed robust vimentin staining. It has been proposed that HIF‐1α drives EMT through the direct activation of master regulatory transcription factors Snail, Slug, or Twist1.^[^ ^39^, ^40^, ^41^, ^42^ ^]^ Other reports, however, have shown that hypoxia triggers EMT by increasing expression of TGF‐β1.^[^ ^43^, ^44^ ^]^ We found that treatment with DPCA was only associated with a significant increase in mRNA for *SNAI2* (encoding for Slug protein) and *TWIST1* (Figure 5B). Although *SNAI1* and *TGFB* expression were slightly elevated, it was not statistically different from untreated controls (*p* > 0.05). In colon tissue samples from DPCA‐treated mice, we also did not observe a significant increase in TGF‐β1 production but did note increased nuclear staining for Snail and Twist1 specifically co‐localizing to the epithelium (Figure S7, Supporting Information).

### 2.4 DPCA Improves Barrier Function in Caco‐2 Cell Monolayers

**Figure 6:**
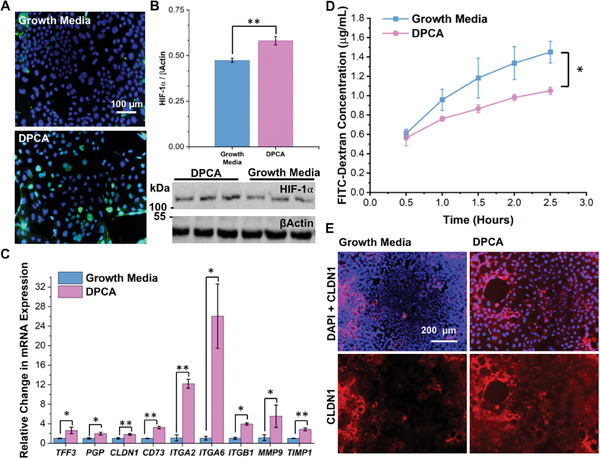
In vitro activity of DPCA was studied in the human intestinal cell line, Caco‐2. After 4‐hour incubation with drug, A) increased HIF‐1α staining (green, DAPI counter stain) was seen and B) verified through western blotting. C) HIF‐1α‐induced expression of barrier‐specific and anti‐inflammatory genes were verified at the mRNA level in DPCA‐treated cells. D) Transcriptomic changes improved barrier function in Caco‐2 monolayers as determined by a FITC‐dextran permeability assay. E) In these monolayers, greater expression of tight junction protein, claudin‐1 (CDN1, red with DAPI counterstain) was also noted following 24‐hour treatment with DPCA (Student's *t*‐test, *N* = 2–3 in C and 3 in D; ^*^ *p* < 0.05, ^**^ *p* < 0.01).

To begin to assess the clinical utility of DPCA in treating human IBD, we explored the drug's effects on the human colonic cell line, Caco‐2. After 4‐hour incubation with free DPCA, we observed elevated expression of HIF‐1α (**Figure** **6**A,B). In agreement with our previous findings, we did not observe any change in HIF‐2α levels, or expression of *DMT1*, a known genetic target of HIF‐2α but not HIF‐1α (Figure S8, Supporting Information)^[^ ^45^ ^]^ 24 h of incubation with DPCA also led to increased mRNA expression of HIF‐1α gene targets involved in barrier function (*TFF3*, *P‐GP*, *CLDN1*), anti‐inflammatory signaling (*CD73*), cell migration (*ITGA2*, *ITGA6*, *ITGB1*), and tissue remodeling (*MMP9*, *TIMP1*) (Figure 6C).

After verifying the bioactivity of DPCA in human cells, we tested the drug's effects on barrier function. To mimic the intestinal epithelium, we cultured Caco‐2 cells on porous transwell inserts. Under these conditions, Caco‐2 cells are known to spontaneously differentiate to form polarized monolayers that resemble the intestinal barrier.^[^ ^46^, ^47^ ^]^ We then treated monolayers with normal growth media or media containing DPCA for 24 h. To evaluate changes in barrier function, apical media was replaced with a buffered solution of FITC‐dextran and the basolateral compartment was filled with fresh buffer. At designated times points, we sampled the basal buffer to quantify the movement of FITC‐dextran across the cell layer. As seen in Figure 6D, monolayers treated with DPCA prevented FITC‐dextran diffusion to a greater extent than growth media controls. This is likely due to increased expression of tight junction proteins, such as Claudin‐1 (*CLDN1*), which are known to be regulated by HIF‐1α (Figure 6C,E).

## 3 Discussion

Structural repair of the intestinal epithelium is critical to restoring barrier function in patients with IBD. Despite its correlation to disease remission, however, histological healing is often ignored as a therapeutic outcome.^[^ ^2^, ^48^ ^]^ While several cell‐based therapies have been explored in preclinical research, clinical scale‐up and standardization of these techniques, combined with concerns of allogenic cell immunogenicity, have limited their wide‐spread implementation.^[^ ^49^, ^50^, ^51^, ^52^, ^53^ ^]^ Thus, there exists a dire unmet need for pro‐regenerative IBD therapies. Ideally, such a treatment would not only mitigate inflammation, but induce rapid re‐epithelialization of the GIT. To fulfill this unmet need, in this study we attempt to leverage the PHD inhibitor molecule DPCA, which was previously shown to induce regeneration in adult mice through transient stabilization of the transcription factor HIF‐1α.^[^ ^7^, ^8^, ^9^, ^10^ ^]^ Given past work on DPCA‐induced regeneration and the role of HIF‐1α in intestinal homeostasis, we hypothesized that transient delivery of DPCA would expedite colon repair and disease recovery in experimental colitis.

When administered before the onset of disease, we found that two injections of DPCA were able to reduce weight loss in mice exposed to drinking water containing 1.5% DSS. DPCA's ability to protect against DSS‐induced colitis is likely a direct effect of HIF‐1α stabilization in the colon, which serves to increase mucus production and the expression of barrier‐protective genes. We also found that delivery of DPCA after the onset of disease was able to accelerate weight recovery and epithelial repair. This effect was accompanied by a drop in pro‐inflammatory cytokine expression, possibly due to enhanced homing of Tregs to the site of injury. Histological healing of the colon lining was likely driven by a HIF‐1α‐induced EMT, which was verified in drug‐treated epithelial cells. Previously, we demonstrated DPCA's ability to promote regeneration of ear hole wounds and tissue lost to periodontitis.^[^ ^7^, ^8^, ^9^, ^10^ ^]^ This work is distinct, as it illustrates the broad utility of DPCA by validating its efficacy in a new organ system (intestines) and pathology (chemically‐induced wounding and colitis). While select studies have explored the use of PHD inhibitors in experimental colitis, all studies to date have only shown a protective effect (i.e., treatment was given before tissue damage). These drugs were also delivered via multiple injections at a 24x higher dosage than the amount of DPCA used in this study.^[^ ^23^ ^]^ Here we also show that low doses of DPCA promote healing of tissue following active disease, which better recapitulates clinical treatment regimens. By transiently upregulating HIF‐1α pharmacologically through the controlled release delivery system, we believe that we mitigate many of the potentially deleterious effects of long‐term HIF‐1α stabilization, such as inflammation.^[^ ^54^ ^]^ We also believe that the specificity of DPCA for HIF‐1α over HIF‐2α may also increase the safety of our therapy.^[^ ^27^ ^]^

Overall, this study suggests that short‐term treatment with DPCA may accelerate ulcer repair and clinical symptoms of experimental colitis. Future work is needed to elucidate the full biological phenomena driving this effect, since HIF‐1α is thought to regulate over 100 genetic targets.^[^ ^7^, ^12^ ^]^ Such studies may be performed using ex vivo organoids, as new models are created to recapitulate clinical disease.^[^ ^55^, ^56^, ^57^, ^58^ ^]^ By identifying downstream pathways, new therapies may be developed to accelerate wound repair. To improve therapeutic efficacy, drug localization to the GIT may also be of interest in the design of future DPCA carriers.^[^ ^59^ ^]^ We believe that DPCA may be combined with existing anti‐inflammatory agents to increase the likelihood of disease remission or may one day replace existing standards of care as a stand‐alone therapy for IBD.

## 4 Experimental Section

### Mice

Eight‐week old female C57BL/6J mice were purchased from the Jackson Laboratory and used in all experiments. Mice were maintained in groups of five in ventilated cages under specific pathogen‐free conditions on a standard 12‐h light/dark cycle. Food and water (with or without DSS) were provided ad libitum. All animal experiments were reviewed and approved by the Institutional Animal Care and Use Committee of the University of California, Berkeley (AUP‐2021‐08‐14573) and were performed in compliance with institutional, state, and federal policies.

### Induction of Acute Colitis Using DSS

For DSS and DSS, DPCA treatment groups (*N* = 5/group), cage water was replaced with sterile water containing 1.5% DSS (MW 36k to 50 kDa, MP Biomedicals CAS: 9011‐18‐1). Water consumption was monitored daily and replenished every 3 days. Mice were weighed daily and inspected for signs of disease onset (increases in bowel movement frequency, stool softening, appearance of blood on fur or rectum). On day 10, DSS water was removed and replaced with normal drinking water. Mice were then scarified on day 12 via carbon dioxide inhalation and cervical dislocation. At the time of sacrifice, colons were resected from the cecum to the anus and measured. Tissue was then collected immediately and preserved for mRNA extraction or histology. Normal drinking water was maintained over the course of the experiment for healthy controls.

### Cell Culture

C57BL/6 mouse primary colonic epithelial cells (pMECs) were purchased from Cell Biologics (Cat: C57‐6047) and grown in Complete Epithelial Cell Medium (Cell Biologics Cat: M6621) containing 10% fetal bovine serum, 100IU/mL Penicillin‐streptomycin, 0.1% epidermal growth factor, and 0.1% Insulin‐Transferrin‐Selenium. All tissue culture flasks and well plates were pre‐coated with gelatin (Cell Biologics Cat: 6950). Cells used in the described experiments were derived from less than 10 rounds of passaging. Human epithelial Caco‐2 cells (ATCC HTB‐37) were maintained in complete high glucose DMEM media (with l‐glutamine and sodium pyruvate) supplemented with 10% fetal bovine serum, 100IU/mL Penicillin‐streptomycin, 100 um HEPES buffer, and 1x Nonessential Amino Acid. RAW246.7 murine macrophages (ATCC TIB‐71) were grown in high glucose DMEM media (with l‐glutamine and sodium pyruvate) supplemented with 10% fetal bovine serum, 100IU/mL Penicillin‐streptomycin, and 100 uM HEPES. All cells were grown at 37 °C, 5% CO_2_, and 21% O_2_ with frequent media changes. Subculturing for all cell types was performed at 80% confluence using 0.05% trypsin‐EDTA. For DPCA‐treated samples, RAW246.7 macrophages and Caco‐2 cells were treated with growth media containing 30 µg mL^−1^ DPCA for 4 or 24 h. For pMECs, cells were treated for 4 or 48 h with 50 µg mL^−1^ DPCA. Caco‐2 hypoxia cultures were grown in 1% O_2_ for 6 h.

### Therapeutic Intervention with DPCA

For preventative treatment with DPCA (DPCA‐PT), a 5 µL injection of the PEG‐based hydrogel was administered subcutaneously in the back of the neck on day −2 before DSS exposure. On day 0, mice were then exposed to DSS at 1.5 wt.%. On day 5, mice from all treatment groups (preventative DPCA‐PT or restorative DPCA‐RT) received a 5 µL injection of the DPCA gel. All injections were performed with 23‐gauge needle loaded with pre‐warmed gel.

### In Vivo Imagining of ROS Activity

At day 9, mice from treatment and control groups were placed under anesthesia following exposure to 1 to 3% isoflurane. 100 µL of L‐012 (20 mm, FUJIFILM Wako Chemicals U.S.A. Corp, Cat: 120–04891) prepared in sterile 1x PBS, was then administered via intraperitoneal injection using a 25‐gauge needle. Mice were then loaded into a PerkinElmer's IVIS Spectrum in vivo imaging system, where isoflurane exposure was maintained via a nose cone. After 5 min, chemiluminescent images were obtained. Mice were then placed back in their home cages for recovery. All image analysis was performed on the IVIS Live Imaging software. Regions of interest (ROI) showing chemiluminescent signal above 1 × 10^4^ radiance were manually selected. Average radiance was then determined and multiplied by ROI area. If multiple ROIs were identified for an animal, total radiance was reported as the sum of all scaled radiance values.

### Quantitative Real‐Time PCR (RT‐qPCR)

Following sacrifice on day 12, mouse colons were immediately harvested from the cecum to anus and placed in cold PBS. Colons were flushed several times with cold PBS to removal stool and contaminants, and then divided into three equal sections beginning immediately below the cecum. The middle region was designated for RT‐qPCR and flash frozen on dry ice. Tissue samples were stored at −80 °C until further use. For mRNA extraction, 15 mg of frozen tissue was transferred to lysis buffer supplied from a Qiagen mRNeasy kit (Cat: 74104) supplemented 0.1% 2‐mercaptoethanol and homogenized using a POLYTRON PT1200 homogenizer for 30 s. mRNA was then isolated using spin column separation according to the manufacturer's instructions. Total mRNA and purity were determined using a Take3 Micro‐Volume Plate on a BioTek SynergyH1 Microplate Reader and converted to cDNA using Maxima H Minus cDNA Synthesis Master Mix (Thermofisher Scientific Cat: M1661). For in vitro cell cultures, cells were grown at a density of 2 × 10^5^ cells/well in six‐well plates. Cell lysis was performed directly in well plates and mRNA was again extracted using a Qiagen mRNeasy kit and converted to cDNA using cDNA using Maxima H Minus cDNA Synthesis Master Mix. All RT‐qPCR was performed with PowerUp SYBR Green Master Mix (Thermofisher Scientific Cat: A25741) on Quantagene q225 qPCR System. Gene‐specific primers were supplied via Integrated DNA Technologies and can be found in Tables S1 and S2 (Supporting Information). All analysis was done using the ΔΔCt approach between treatment and untreated controls and are based on samples. One tissue sample from the DPCA‐PT group was excluded from analysis due to an error in handling.

### Immunocytochemistry

Cells were seeded at a density of 10 000 cells per well in a 96‐well plate and grown overnight. Cells were then treated with DPCA (30 to 50 µg mL^−1^) and incubated at 37 °C, 5% CO_2_ for 4, 24, or 48 h. At the conclusion of treatment, media was removed, and all wells were washed three times with warm PBS. Cells were fixed with 4% PFA for 15 min at room temperature and washed again. Cells were then permeabilized with 0.1% Triton solution prepared in 5% goat serum for 20 min at room temperature. After several washes with 5% goat serum, 100 µL of the solution was left in the plate to block for 1 h at room temperature. Goat serum was then removed and replaced with 50 µL of the primary antibody solution, diluted in 5% goat serum (1:500 anti‐HIF‐1α [EP1215Y] Abcam ab51608; 1:200 anti‐Claudin 1 Thermofisher 51–9000; 1:200 ant‐Vimentin (EPR3776) Abcam ab92547). The plate was incubated at 4 °C overnight and then washed three times with PBS. Secondary antibodies (Alexa Flour 568‐ or Alexa Flour 488‐goat anti‐rabbit) diluted 1:300 in PBS were then added to each well and incubated at RT for 1 h (in dark). After washing, cells were counterstained with antifade mountant with DAPI (Molecular Probes) and imaged using a Keyence BZX fluorescent microscope. In some experiments, actin staining was performed using ActinGreen 488 ReadyProbes Reagent (ThermoFisher Cat: R37110) according to the manufacturer's instructions.

### Histology and Immunofluorescence Histochemistry

At sacrifice, mouse colons were resected and measured for total length from below the cecum to rectum. The tissue was then divided into three equal sections. The upper section was immediately fixed in 4% PFA, dehydrated, cleared in xylene, and embedded in paraffin. The lower section of the colon was cut longitudinally to expose the lumen and then Swiss rolled from the rectum. The tissue was secured in a cassette fixed in 4% PFA, dehydrated, cleared in xylene, and embedded in paraffin. Blocks were sectioned at 5‐µm thickness to expose the cross‐section of the colon or roll. The sections were then stained with Thermo Scientific Shandon Gill Hematoxylin 2 (Cat: 6765007) and Eosin Y (Sigma Cat: HT110216), Masson's trichrome stain (Abcam Cat: ab150686), or Alcian Blue (Poly Scientific R&D Corp. Cat: s111a) with Nuclear Red Fast (Abcam Cat: AB246831). For grading, a blind volunteer reviewed 4 H&E images per mouse at three separate times to eliminate bias and variance in grading. Images were graded 0 to 3 using the rubric established by Koelink, et. al. and shown in Table 1.^[^ ^29^ ^]^ One tissue sample from the DPCA‐PT group was excluded from analysis due to an error in handling. For immunofluorescence histochemistry, the sections were rehydrated and cleared with xylene, and then subjected to heat‐induced antigen retrieval with sodium‐citrate buffer (pH 6) or TRIS‐EDTA (pH 9). Slides were permeabilized, blocked with goat serum, and stained with primary antibodies (1:400 anti‐HIF‐1α [EP1215Y] Abcam ab51608; 1:200 anti‐Claudin 1 Thermofisher 51–9000; 1:400 anti‐Fox3P Novus Biologicals NB10039002; 1:250 anti‐TFF3 [EPR3974] Abcam ab108599; 1:250 anti‐p‐GP [EPR10364‐57] Abcam ab170904; 1:100 anti‐HIF‐2α Novus Biologics NB100‐122; 1:150 anti‐TWIST1 Thermofisher PA549688; 1:200 anti‐Snail Invitrogen PA549688; 1:200 anti‐vimentin (EPR3776) Abcam ab92547) followed by the appropriate secondary antibodies (Alexa Flour 568‐ or Alexa Flour 488‐goat anti‐rabbit). Slides were mounted with cover slips using Vectasheild mounting medium with DAPI or ProLong Diamond Antifade mountant with DAPI and imaged on a Keyence BZX fluorescent microscope. To quantify HIF‐1α staining, the study randomly imaged three to five colon slices for every mouse in treatment or control groups at 20x magnification. Using ImageJ, the raw integrated density across each image was determined. To account for differences in cell density and tissue size, the raw integrated densities were normalized to DAPI area staining.

### Western Blot

For Caco‐2 samples, 3 × 10^6^ cells were plated in 6‐well plates and grown for two days in normal growth media. On the day of the experiment, media was replenished in all control wells, or replaced with media containing 40 µg mL^−1^ DPCA for drug‐treated samples. After 4 h, all media was removed, and the cells were washed with cold PBS. Cells were then lysed with 100 µL of Pierce RIPA buffer (Thermofisher Scientific Cat: 89900) with Halt protease inhibitor cocktail (Thermofisher Scientific Cat: 1862209) and total protein content in the lysate was determined using a BCA assay (Thermofisher Scientific Cat: 23227). Seven microgram of protein was then loaded into Bolt Bis‐Tris Plus Mini Protein Gels (Thermofisher Scientific Cat: NW04125), electrophoresed in MOPS running buffer at 200 V for 35 min, and electrotransferred onto a Power Blotter nitrocellulose membrane (Thermoscientific Cat: PB7320). The membrane was washed, blocked with 5% BSA, and incubated with primary antibodies (1:1000 anti‐HIF1α Thermofisher Scientific MA1‐516; 1:1000 anti‐βActin Cell Signaling Technology 4967) overnight at 4 °C. Membranes were then washed and incubated with horseradish peroxidase‐linked secondary antibodies (1:5000 Goat anti‐rabbit Prometheus Protein Biology Product 20–303; Goat anti‐mouse Prometheus Protein Biology Product 20–304) for 1 h. Immediately before imaging, blots were developed using the Thermo Scientific Pierce ECL Western Blotting Substrate (Cat: PI32109). Imaging was then performed on an Invitrogen iBright FL1000 system. For tissue samples, 5 mg of whole colon tissue was homogenized in 300 µL of RIPA buffer with protease inhibitor cocktail using stainless steel beads and vortexing. The resulting protein lysate was quantified and used as described above. ImageJ was used to quantify protein band signal, by determining the mean grayscale value of each band, normalized to βActin.

### FITC‐Dextran Permeability Studies with Caco‐2 Cells

Caco‐2 cells were seeded onto transwell inserts (0.4 µm pore size, 12‐well, PET (Corning Cat: 353180) at a density of 2 × 10^5^ cells/cm^2^ and maintained for 18 days with frequent media changes to induce differentiation. Maturity was assessed using an EVOM2 Trans Epithelial Electrical Resistance (TEER) meter with STX2 electrode. Only cell layers exhibiting a TEER greater than 2000 Ω × cm^2^ were used in permeability assays. For DPCA‐treated samples, media containing 20 µg mL^−1^ DPCA was added to the upper and lower chamber of the transwell insert. After 24 h, media was removed, and all cells were washed with warm PBS. Drug‐treated cells and growth media controls (*n* = 3) were then equilibrated with HBSS solution for 1 h at 37 °C, 5% CO_2_. At *t* = 0, a solution of FITC‐dextran (1 mg mL^−1^, MW 4 kDa, Sigma Aldrich Cat: 46944) prepared in Hank's Balanced Salt Solution (HBSS), was then added to the upper compartment of the transwell insert. The lower compartment was refreshed with HBSS and the plate was placed back in the incubator. At 30‐minute intervals, buffer was removed from the lower chamber and replaced with fresh HBSS. FITC‐dextran concentration in the basolateral compartment was determined by reading the fluorescence of the sample at 520, 488 nm excitation on a BioTek SynergyH1 Microplate Reader. Fluorescence intensity was converted to concentration using a standard curve based on FITC‐dextran solutions prepared at known concentrations.

### Statistical Analysis

A two‐tailed Student's *t‐test* was used for two‐group comparisons in Excel. *p*‐values < 0.05 were statistically significant. All error bars are based off standard deviation between biological replicates unless otherwise specified.

## Conflict of Interest

Two of the authors (PBM and EHK) have formed a company that is commercializing the drug delivery technology reported in this study.

## Supporting information

Supporting Information

## Data Availability

The data that support the findings of this study are available in the supplementary material of this article.
