# Supplementary material for: A Pro‐Regenerative Supramolecular Prodrug Protects Against and Repairs Colon Damage in Experimental Colitis
Source: Adv Sci (Weinh). 2024 Jan 21;11(13):2304716. doi: 10.1002/advs.202304716 (PMC10987129; doi:10.1002/advs.202304716)
Supplement: Supplementary file 1 — Supporting Information [file ADVS-11-2304716-s001.pdf]

ADVANCED SCIENCE
Open Access

# Supporting Information

for *Adv. Sci.*, DOI 10.1002/advs.202304716

A Pro-Regenerative Supramolecular Prodrug Protects Against and Repairs Colon Damage in Experimental Colitis

*Kelsey G. DeFrates, Elaine Tong, Jing Cheng, Ellen Heber-Katz and Phillip B. Messersmith**

**Supplementary Information For:**

# A pro-regenerative supramolecular prodrug protects against and repairs colon damage in experimental colitis

Kelsey G. DeFrates, Elaine Tong, Jing Cheng, Ellen Heber-Katz, Phillip B. Messersmith*

Department of Bioengineering, University of California, Berkeley, Berkeley, CA 94720, USA. (K.G.D, E.T., J.C., P.B.M.)
Lankenau Institute for Medical Research, Wynnewood, Pennsylvania, 19096, USA
Department of Materials Science and Engineering, University of California, Berkeley, Berkeley, CA 94720, USA (E.H.K.)
Materials Sciences Division, Lawrence Berkeley National Laboratory, Berkeley, CA 94720, USA (P.B.M)
*Correspondence: University of California, Berkeley, 210 Hearst Mining Building Berkeley, CA 94720-1760, USA; E-mail: philm@berkeley.edu

Preparation of Prodrug Hydrogel

PEG-DPCA hydrogels were prepared as previously described.[1] Briefly, methoxy PEG (MW 750 g/mol, P7) or telechelic PEG (MW 8000 g/mol, P80) were activated via TEMPO-mediated oxidation and end functionalized with 2-amino-2- (hydroxymethyl)-1,3-propanediol via HBTU-mediated coupling. DPCA was then conjugated to the terminal hydroxyls of the PEG chains via 1,1′-Carbonyldiimidazole-activated esterification to yield fiber-forming low molecular prodrugs (P7D3) or high molecular weight, telechelic bridging molecules (P80D6). To generate hydrogels, P7D3 fibers were formed by first dissolving the prodrugs in DMSO at 20 mg/mL and diluting 1:10 with DI $H_2O$. The solution was then dialyzed for 48 hours (4x bath changes) in a 5K MWCO cassette to remove free drug. Aqueous fiber suspensions were lyophilized and resuspended in sterile water at 15 mg/mL. Lyophilized P80D6 was then dissolved in the P7D3 solution to 85 mg/mL by vigorous vortexing and heating. Final gels were prepared and stored under sterile conditions. Successful conjugation of DPCA to PEG was verified via using solution state 1H-NMR in DMSO-d6 on a Bruker Avance 400 console with Oxford Instruments 9.4 T magnet (AVB-400 MHz) NMR instrument (**Figure S1**).

ELISA

RAW246.7 murine macrophages were seeded at a density of $9x10^5$ cells/well in 6 well plates. Cells were grown overnight and then treated with growth media containing 1 ug/mL liposaccharide (LPS) from *E. coli* (O111:B4 Sigma Aldrich Cat: L4391) with or without 30 μg/mL DPCA. After 24 hours, media was collected and stored at -80°C until use. The concentration of TNFα in cell culture extracts was then quantified using a mouse anti-TNFα ELISA kit (Millipore Sigma Cat: RAB0477) according to the manufacturer's instructions. Samples were diluted 1:50 to allow for adequate detection and compared to a standard curve prepared from reconstituted mouse TNFα.

**Table S1**: Primers for RT-qPCR (mouse).

| Gene | Species | F primer | R primer |
|---|---|---|---|
| *EEF2* | Mouse (Housekeeping) | CCGACTCCCTTGTGTGCAA | AGTTCAGGTCGTTCTCAGAGAG |
| *ACTB* | Mouse (Housekeeping) | GTGACGTTGACATCCGTAAAGA | GCCGGACTCATCGTACTCC |
| *TNFA* | Mouse | CAGGCGGTGCCTATGTCTC | CGATCACCCCGAAGTTCAGTAG |
| *TGFB* | Mouse | CCACCTGCAAGACCATCGAC | CTGGCGAGCCTTAGTTTGGAC |
| *IL22* | Mouse | ATGAGTTTTTCCCTTATGGGGAC | GCTGGAAGTTGGACACCTCAA |
| *CLDN1* | Mouse | TGCCCCAGTGGAAGATTTACT | CTTTGCGAAACGCAGGACAT |
| *IL10* | Mouse | CTTACTGACTGGCATGAGGATCA | GCAGCTCTAGGAGCATGTGG |
| *FOXP3* | Mouse | CACCTATGCCACCCTTATCCG | CATGCGAGTAAACCAATGGTAGA |
| *CXCR4* | Mouse | GACTGGCATAGTCGGCAATG | AGAAGGGGAGTGTGATGACAAA |
| *MUC2* | Mouse | AGGGCTCGGAACTCCAGAAA | CCAGGGAATCGGTAGACATCG |
| *VIM* | Mouse | CGTCCACACGCACCTACAG | GGGGGATGAGGAATAGAGGCT |
| *CDH1* | Mouse | CAGTTCCGAGGTCTACACCTT | TGAATCGGGAGTCTTCCGAAAA |
| *SNAI1* | Mouse | CACACGCTGCCTTGTGTCT | GGTCAGCAAAAGCACGGTT |
| *SNAI2* | Mouse | CAGCGAACTGGACACACACA | ATAGGGCTGTATGCTCCCGAG |
| *TWIST1* | Mouse | GGACAAGCTGAGCAAGATTCA | CGGAGAAGGCGTAGCTGAG |

**Table S2:** Primers for RT-qPCR (human).

| Gene | Species | F primer | R primer |
|---|---|---|---|
| *ACTB* | Human (Housekeeping) | CATGTACGTTGCTATCCAGGC | CTCCTTAATGTCACGCACGAT |
| *TFF3* | Human | CCAAGCAAACAATCCAGAGCA | GCTCAGGACTCGCTTCATGG |
| *PGP* | Human | TTGCTGCTTACATTCAGGTTTCA | AGCCTATCTCCTGTCGCATTA |
| *DMT1* | Human | TGGAGATCATGGGGAGTCTG | AAGAAAACCTGGTCCGGTGAA |
| *MMP9* | Human | TGTACCGCTATGGTTACACTCG | GGCAGGGACAGTTGCTTCT |
| *TIMP1* | Human | CTTCTGCAATTCCGACCTCGT | ACGCTGGTATAAGGTGGTCTG |
| *CLDN1* | Human | CCTCCTGGGAGTGATAGCAAT | GGCAACTAAAATAGCCAGACCT |
| *CD73* | Human | GCCTGGGAGCTTACGATTTTG | TAGTGCCCTGGTACTGGTCG |
| *ITGA2* | Human | CCTACAATGTTGGTCTCCCAGA | AGTAACCAGTTGCCTTTTGGATT |
| *ITGB1* | Human | CCTACTTCTGCACGATGTGATG | CCTTTGCTACGGTTGGTTACATT |
| *ITGA6* | Human | ATGCACGCGGATCGAGTTT | TTCCTGCTTCGTATTAACATGCT |

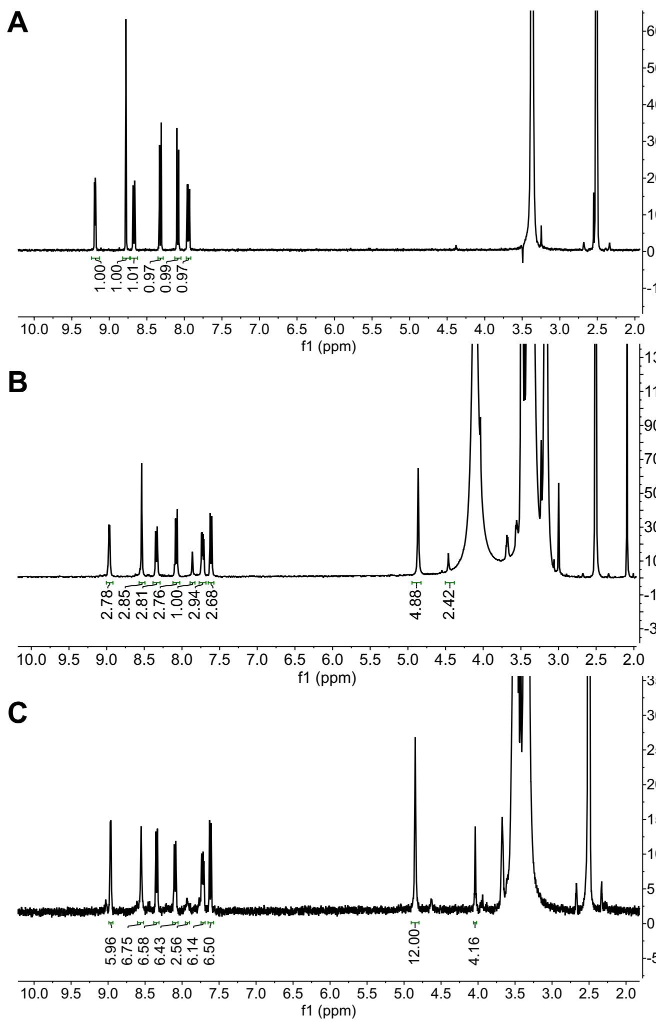


**Figure S1.** 1HNMR analysis of free DPCA (A), PEG7D3 (B), and P80D6 (C).

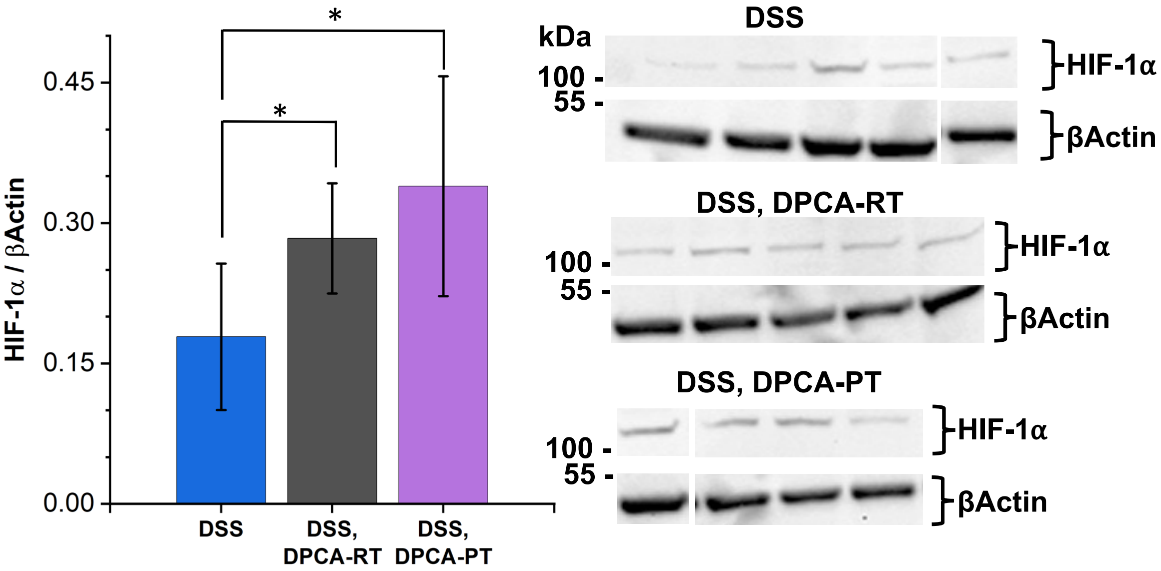


**Figure S2.** Western blot analysis of colon tissue samples from DSS only control mice, and those treated with DPCA. An observable increase in staining is seen in DSS, DPCA-RT group, and a significant increase is seen in DSS, DPCA-PT. (student's t-test, N=5 (DSS and DSS, DPCA-RT, or 4 DSS, DPCA-PT, * $p < 0.05$).

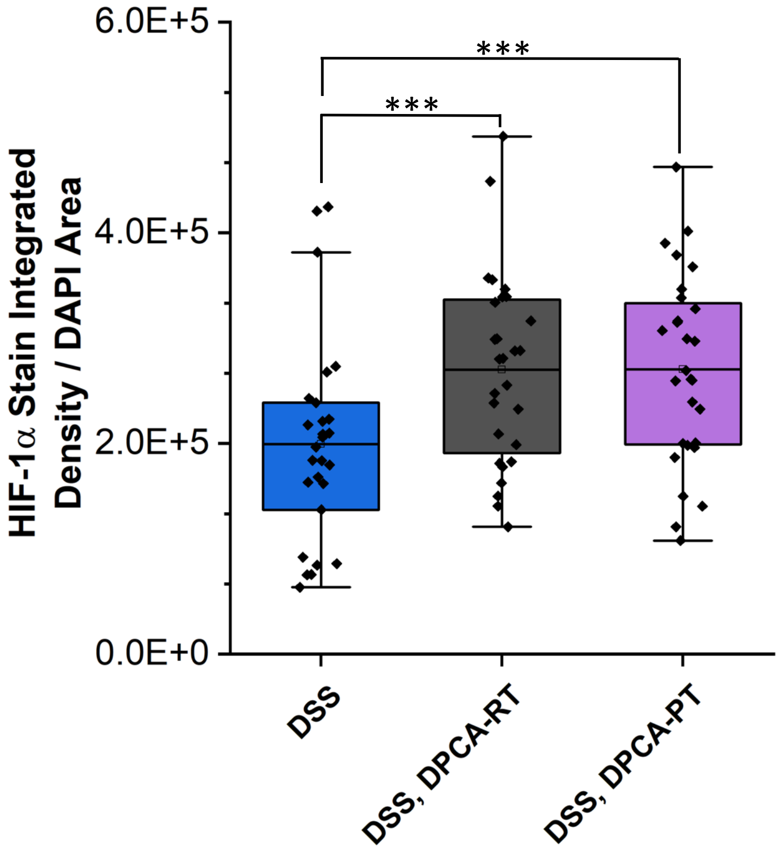


**Figure S3.** The integrated density for HIF-1α immunofluorescence staining is given for DSS only controls and DPCA treatment groups. (Student's t-test N = 3 to 5 images per animal (N=5), *** $p < 0.001$).

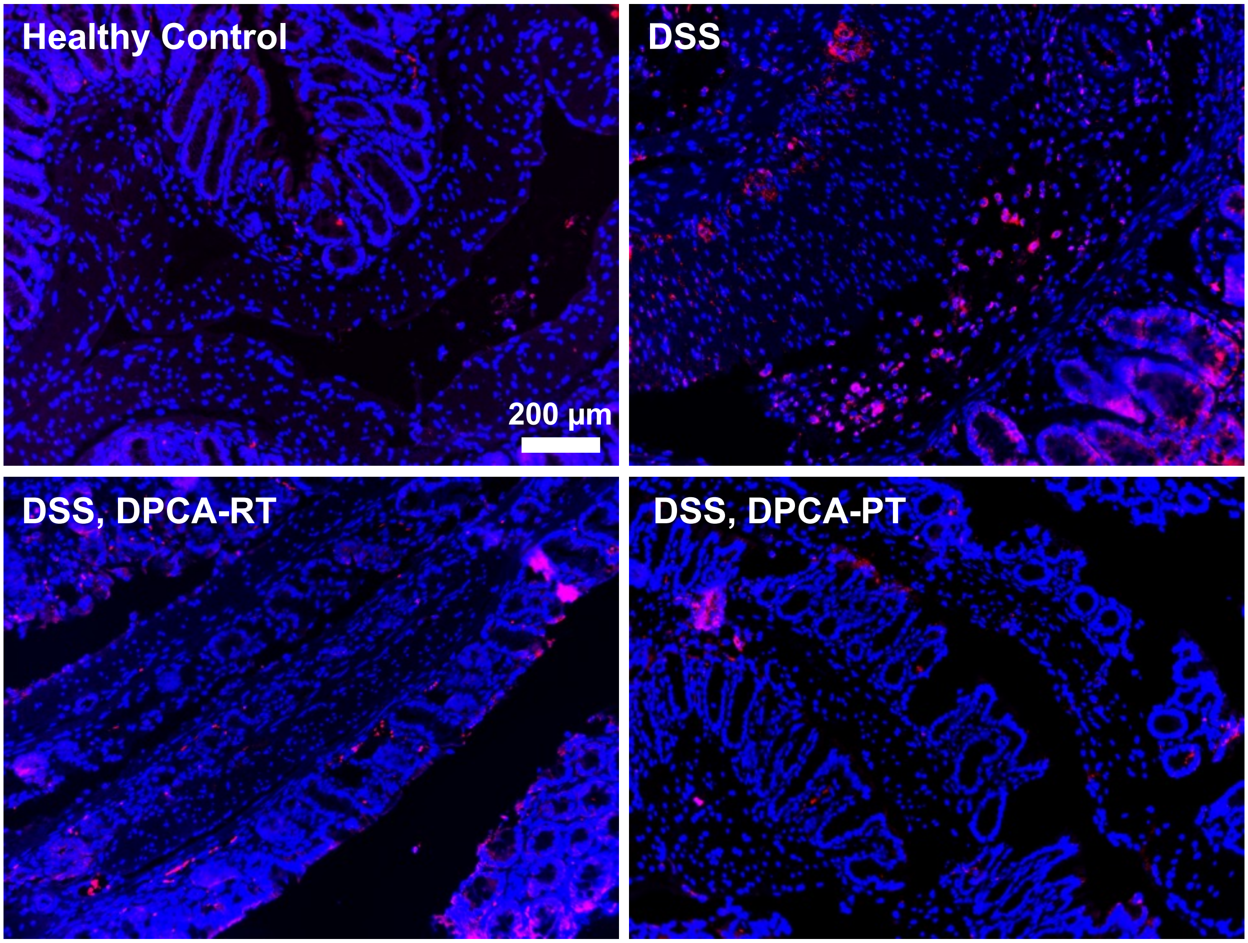


**Figure S4.** Immunofluorescence staining for HIF-2$\alpha$ in healthy, non-treated, and DPCA-treated mice (red, DAPI counterstain).

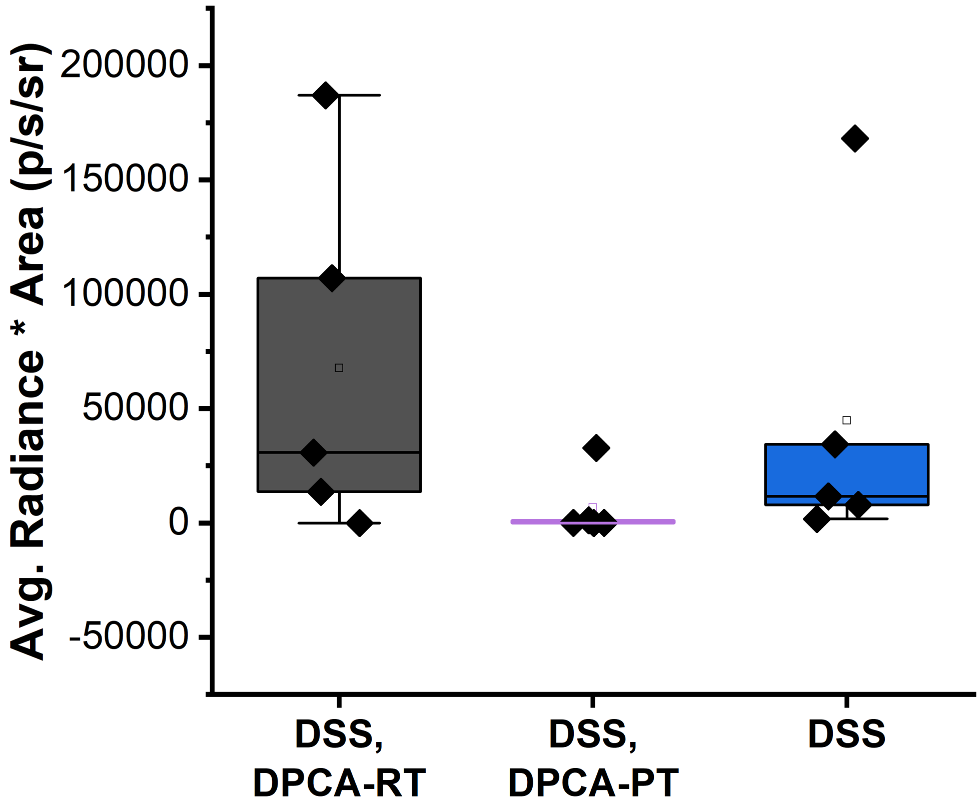


**Figure S5.** Quantitative chemiluminescent data for IVIS study of ROS abundance at day 9 in DPCA-treated and colitic control mice (N = 5).

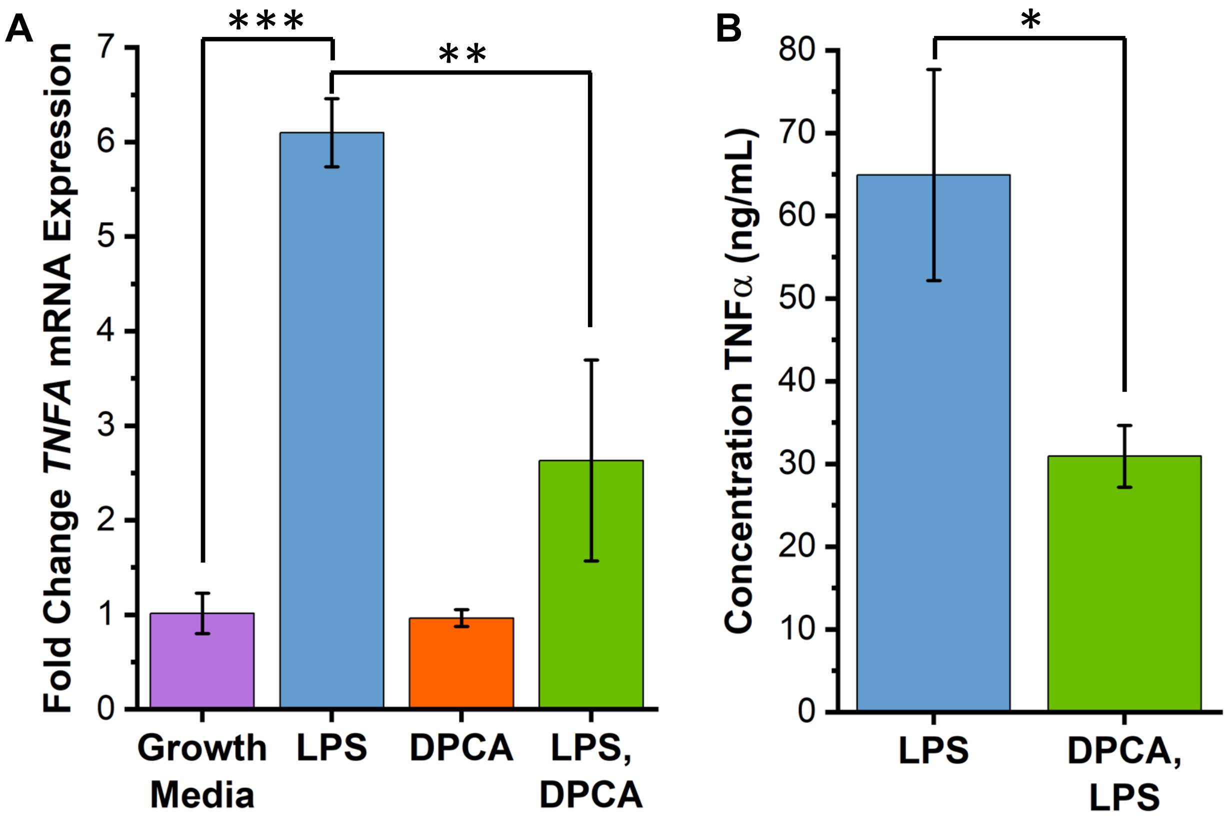


**Figure S6.** Fold change in mRNA expression of TNFA in RAW 246.7 murine macrophages treated with DPCA and/or the endotoxin, liposaccharide (LPS) (A). TNFα protein is quantified in media supernatants from treated and non-treated cell monolayers (B). (Student's t- test, N = 2 to 3, * $p < 0.05$; ** $p < 0.01$; *** $p < 0.001$).

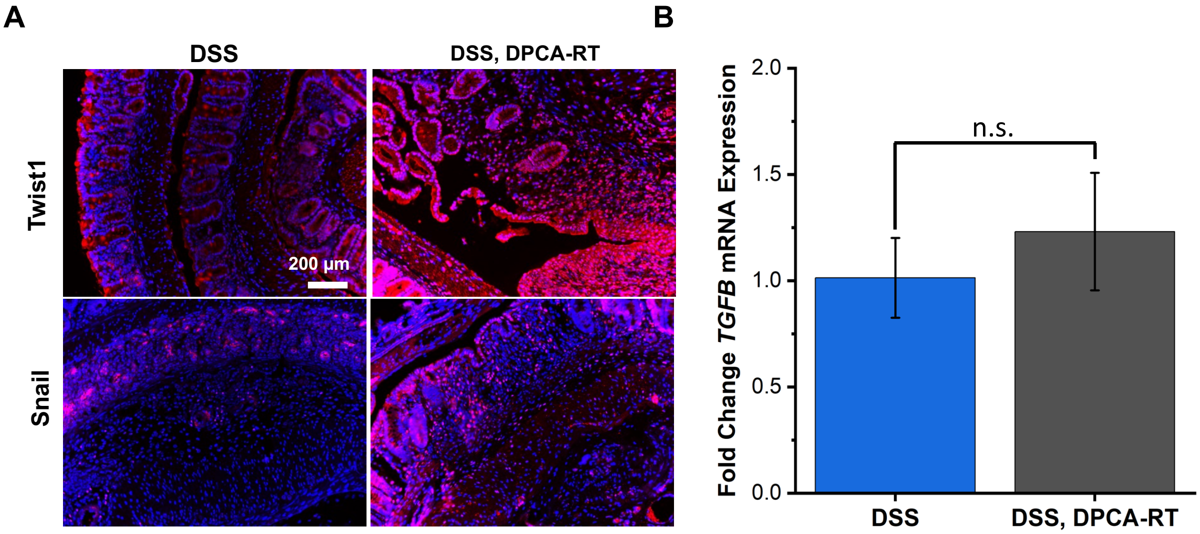


**Figure S7.** Staining for EMT-associated transcription factors, Twist1 and Snail in mouse colon tissue samples (red with DAPI counterstain) (A). mRNA expression of *TGFB* in DPCA-treated mice and non-treatment colitic controls (B). (Student's t-test, N =. 5, n.s. = not significant ($p > 0.05$)).

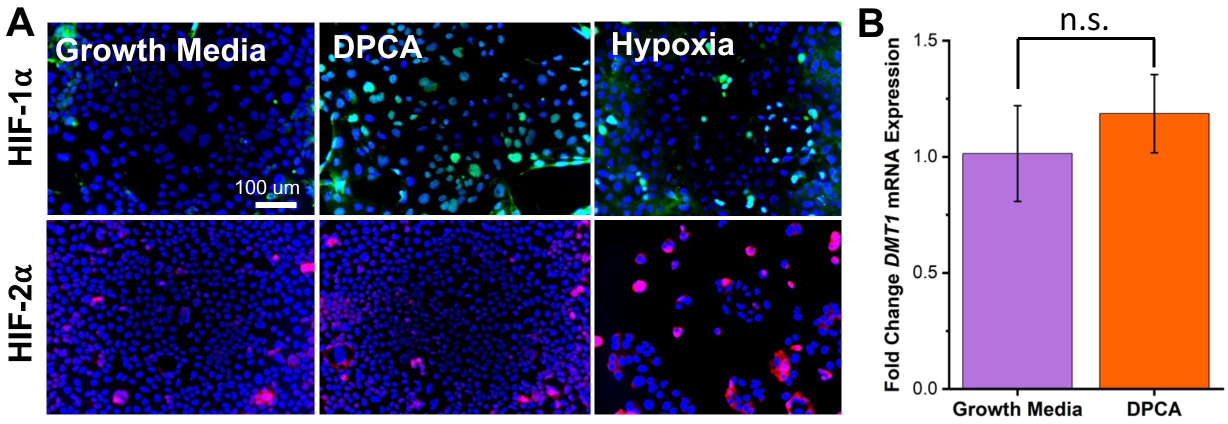


**Figure S8.** Immunofluorescence staining for HIF-1α (green) and HIF-2α (red) in Caco-2 cells grown in 21% oxygen with or without DPCA and under hypoxic conditions (DAPI counterstain) (A). Fold change in mRNA expression of HIF-2α target, *DMT1*, in DPCA-treated or non-treated Caco-2 cells (B) (Student's t-test, N =3, n.s. = not significant ($p > 0.05$)).

**Table S3-5:** Weight change data for Figure 1A.

| Day | No DSS | | | | | Avg | Stdv | p – DPCA-RT | p – DSS | p – DPCA-PT |
|---|---|---|---|---|---|---|---|---|---|---|
| 1 | 0.00 | 0.00 | 0.00 | 0.00 | 0.00 | 0.00 | 0.00 | -- | -- | -- |
| 2 | 2.49 | 1.03 | 3.16 | 4.62 | 0.49 | 2.36 | 1.66 | 0.201 | 0.147 | 0.109 |
| 3 | 1.99 | 2.06 | 0.53 | 4.10 | 0.00 | 1.74 | 1.60 | 0.082 | 0.036 | 0.330 |
| 4 | 1.49 | 3.09 | -0.53 | 1.03 | 0.00 | 1.02 | 1.41 | 0.827 | 0.545 | 0.952 |
| 5 | 2.49 | 5.67 | 2.11 | 1.03 | 0.00 | 2.26 | 2.14 | 0.899 | 0.666 | 0.828 |
| 6 | 5.97 | 2.58 | 5.79 | 3.08 | -1.47 | 3.19 | 3.02 | 0.051 | 0.111 | 0.800 |
| 8 | 5.47 | 5.15 | 3.68 | 3.59 | 1.47 | 3.87 | 1.59 | 0.084 | 0.020 | 0.232 |
| 10 | 3.98 | 7.22 | 5.26 | 4.10 | 1.96 | 4.50 | 1.93 | 1.6E-4 | 0.001 | 0.038 |
| 12 | 5.47 | 5.15 | 10.53 | 7.69 | 1.96 | 6.16 | 3.18 | 0.012 | 5E-4 | 0.008 |

| Day | DSS | | | | | Avg | Stdv |
|---|---|---|---|---|---|---|---|
| 1 | 0.00 | 0.00 | 0.00 | 0.00 | 0.00 | 0.00 | 0.00 |
| 2 | -2.82 | 4.32 | 1.47 | -2.44 | -1.05 | -0.10 | 2.99 |
| 3 | -5.63 | 3.78 | -3.43 | -6.34 | -4.19 | -3.16 | 4.05 |
| 4 | -2.82 | 7.03 | -1.47 | -3.41 | -0.52 | -0.24 | 4.22 |
| 5 | -1.88 | 8.11 | 0.49 | -2.93 | 2.62 | 1.28 | 4.38 |
| 6 | -4.23 | 5.41 | -1.47 | -4.88 | 0.52 | -0.93 | 4.15 |
| 7 | -4.69 | 3.78 | -3.92 | -4.88 | 4.19 | -1.10 | 4.66 |
| 8 | -4.69 | 3.78 | -6.37 | -3.41 | 1.05 | -1.93 | 4.22 |
| 9 | -7.51 | 1.62 | -12.75 | -6.34 | -2.62 | -5.52 | 5.39 |
| 10 | -9.39 | -1.62 | -15.20 | -10.24 | -5.76 | -8.44 | 5.09 |
| 11 | -9.39 | -2.70 | -12.75 | -9.76 | -6.81 | -8.28 | 3.76 |
| 12 | -7.51 | -0.54 | -8.82 | -7.32 | -3.66 | -5.57 | 3.40 |

| Day | DSS, DPCA-RT | | | | | Avg | Stdv | p - DSS |
|---|---|---|---|---|---|---|---|---|
| 1 | 0.00 | 0.00 | 0.00 | 0.00 | 0.00 | 0.00 | 0.00 | -- |
| 2 | 2.06 | 0.47 | 2.39 | 1.03 | -0.51 | 1.09 | 1.18 | 0.432 |
| 3 | 0.52 | -2.33 | 2.87 | -2.58 | -3.57 | -1.02 | 2.65 | 0.351 |
| 4 | 2.58 | -2.79 | 1.44 | 2.06 | 0.51 | 0.76 | 2.13 | 0.649 |
| 5 | 4.12 | -2.33 | 3.83 | 3.61 | 3.06 | 2.46 | 2.70 | 0.623 |
| 6 | 2.06 | -4.19 | 0.00 | -2.58 | 0.51 | -0.84 | 2.51 | 0.968 |
| 7 | 2.06 | -6.51 | 0.96 | -2.58 | -2.55 | -1.72 | 3.39 | 0.816 |
| 8 | 4.64 | -5.12 | 3.83 | -2.06 | -1.53 | -0.05 | 4.15 | 0.497 |
| 9 | 3.61 | -4.65 | 2.87 | -3.61 | -4.08 | -1.17 | 4.05 | 0.188 |
| 10 | -0.52 | -5.58 | -6.22 | -5.67 | -6.63 | -4.92 | 2.50 | 0.203 |
| 11 | 2.06 | -4.65 | -0.48 | -4.12 | -4.59 | -2.36 | 3.02 | 0.025 |
| 12 | 4.12 | -3.26 | 1.44 | -1.55 | -1.02 | -0.05 | 2.88 | 0.024 |

| Day | DSS, DPCA-PT | | | | | Avg | Stdv | p - DSS |
|---|---|---|---|---|---|---|---|---|
| 1 | 0.00 | 0.00 | 0.00 | 0.00 | 0.00 | 0.00 | 0.00 | -- |
| 2 | -0.49 | -0.48 | 2.04 | 2.39 | -0.91 | 0.51 | 1.57 | 0.696 |
| 3 | -1.95 | 1.92 | 1.02 | 3.83 | -5.00 | -0.04 | 3.47 | 0.226 |
| 4 | 0.98 | 0.96 | 1.53 | 5.26 | -3.18 | 1.11 | 3.00 | 0.576 |
| 5 | 0.00 | 0.96 | 4.08 | 7.18 | 0.91 | 2.63 | 2.98 | 0.586 |
| 6 | -0.49 | 2.40 | 3.06 | 7.18 | 1.36 | 2.70 | 2.84 | 0.145 |
| 7 | 0.00 | -0.96 | 4.08 | 7.18 | 3.18 | 2.70 | 3.27 | 0.174 |
| 8 | -0.49 | 0.00 | 4.08 | 5.74 | 0.91 | 2.05 | 2.72 | 0.114 |
| 9 | 0.00 | -0.96 | 4.08 | 7.18 | 3.18 | 2.70 | 3.27 | 0.020 |
| 10 | -0.98 | -0.96 | 3.06 | 4.31 | -0.45 | 1.00 | 2.50 | 0.006 |
| 11 | 0.00 | -0.48 | 1.02 | 5.74 | 0.45 | 1.35 | 2.52 | 0.001 |
| 12 | -1.95 | 0.96 | -4.08 | 3.35 | -0.91 | -0.53 | 2.83 | 0.034 |

**Table S6:** Colon length measurements for Figure 1B.

| | Healthy | DSS | DSS, DPCA-RT | DSS, DPCA-PT |
|---|---|---|---|---|
| | 8.89 | 6.46 | 8.82 | 8.80 |
| | 9.05 | 7.04 | 8.56 | 8.12 |
| | 9.40 | 7.26 | 8.87 | 8.88 |
| | 9.17 | 6.89 | 9.33 | 8.79 |
| | 8.98 | 6.47 | 8.7 | 9.04 |
| | 9.10 | 6.82 | 8.86 | 8.73 |
| | 0.20 | 0.35 | 0.29 | 0.35 |
| p-value vs. DSS | 1.5E-6 | -- | 8.9E-6 | 2.8E-5 |
| p-value vs. Healthy | -- | 1.5E-6 | 0.16 | 0.074 |

**Table S7-10:** Histological scores of colon tissue for Figures 2B and C.

| **Healthy** | | | | | | | |
|---|---|---|---|---|---|---|---|
| | Inflam. Infil. | Gob. Cell Loss | Crypt Density Red. | Crypt Hyper-plasia | Muscle Thick. | Submuc. Inflam. | Total |
| | 1 | 1 | 1 | 1 | 0 | 0 | 4 |
| | 0 | 2 | 1 | 1 | 1 | 0 | 5 |
| | 0 | 2 | 2 | 1 | 1 | 1 | 7 |
| | 1 | 1 | 0 | 1 | 0 | 1 | 4 |
| | 1 | 0 | 0 | 1 | 0 | 2 | 4 |
| | 2 | 0 | 0 | 1 | 1 | 1 | 5 |
| | 1 | 0 | 0 | 1 | 0 | 1 | 3 |
| | 0 | 0 | 0 | 1 | 0 | 1 | 2 |
| | 0 | 1 | 1 | 1 | 0 | 1 | 4 |
| | 1 | 1 | 1 | 1 | 0 | 0 | 4 |
| | 1 | 1 | 1 | 1 | 1 | 0 | 5 |
| | 0 | 2 | 1 | 1 | 0 | 1 | 5 |
| | 1 | 1 | 1 | 1 | 1 | 0 | 5 |
| | 1 | 3 | 2 | 1 | 2 | 1 | 10 |
| | 1 | 1 | 1 | 1 | 3 | 1 | 8 |
| Average | 0.73 | 1.07 | 0.8 | 1 | 0.67 | 0.73 | 5 |
| Stdev | 0.59 | 0.88 | 0.68 | 0 | 0.9 | 0.59 | 2 |

| DSS | | | | | | | |
|---|---|---|---|---|---|---|---|
| | Inflam. Infil. | Gob. Cell Loss | Crypt Density Red. | Crypt Hyper-plasia | Muscle Thick. | Submuc. Inflam. | Total |
| | 3 | 3 | 0 | 0 | 2 | 2 | 10 |
| | 3 | 3 | 2 | 2 | 3 | 3 | 16 |
| | 3 | 3 | 0 | 0 | 3 | 3 | 12 |
| | 2 | 3 | 1 | 0 | 3 | 2 | 11 |
| | 0 | 1 | 0 | 0 | 1 | 1 | 3 |
| | 3 | 3 | 1 | 0 | 3 | 3 | 13 |
| | 3 | 3 | 3 | 2 | 3 | 3 | 17 |
| | 3 | 2 | 0 | 0 | 2 | 3 | 10 |
| | 3 | 2 | 0 | 0 | 0 | 1 | 6 |
| | 1 | 2 | 0 | 0 | 1 | 1 | 5 |
| | 2 | 3 | 1 | 1 | 3 | 2 | 12 |
| | 3 | 3 | 3 | 3 | 3 | 3 | 18 |
| | 3 | 3 | 1 | 1 | 2 | 3 | 13 |
| | 2 | 3 | 2 | 1 | 3 | 2 | 13 |
| | 0 | 2 | 0 | 0 | 1 | 2 | 5 |
| Average | 2.27 | 2.6 | 0.93 | 0.67 | 2.2 | 2.27 | 10.93 |
| Stdev | 1.1 | 0.63 | 1.1 | 0.98 | 1.01 | 0.8 | 4.53 |
| p-value vs. Healthy | 5.5E-5 | 7.8E-6 | 0.69 | 0.2 | 1.5E-4 | 2.0E-6 | 7.3E-5 |

| DSS, DPCA-RT | | | | | | | |
|---|---|---|---|---|---|---|---|
| | Inflam. Infil. | Gob. Cell Loss | Crypt Density Red. | Crypt Hyper-plasia | Muscle Thick. | Submuc. Inflam. | Total |
| | 1 | 1 | 0 | 1 | 1 | 1 | 5 |
| | 1 | 2 | 0 | 0 | 1 | 2 | 6 |
| | 2 | 2 | 0 | 0 | 0 | 2 | 6 |
| | 1 | 1 | 0 | 1 | 0 | 1 | 4 |
| | 0 | 0 | 0 | 0 | 0 | 0 | 0 |
| | 1 | 2 | 1 | 1 | 1 | 1 | 7 |
| | 1 | 2 | 1 | 0 | 0 | 1 | 5 |
| | 2 | 3 | 0 | 0 | 1 | 1 | 7 |
| | 0 | 1 | 2 | 2 | 1 | 1 | 7 |
| | 2 | 2 | 1 | 1 | 1 | 2 | 9 |
| | 1 | 2 | 0 | 2 | 1 | 1 | 7 |
| | 1 | 3 | 2 | 1 | 1 | 2 | 10 |
| | 2 | 2 | 1 | 0 | 1 | 2 | 8 |
| | 0 | 2 | 1 | 1 | 1 | 1 | 6 |
| | 1 | 1 | 0 | 1 | 0 | 1 | 4 |
| Average | 1.07 | 1.73 | 0.6 | 0.73 | 0.67 | 1.27 | 6.07 |
| Stdev | 0.7 | 0.8 | 0.74 | 0.7 | 0.49 | 0.59 | 2.37 |
| p-value vs. DSS | 1.4E-3 | 2.7E-3 | 0.34 | 0.832 | 1.3E-5 | 5.6E-4 | 9.7E-4 |
| p-value vs. Healthy | 0.17 | 0.04 | 0.45 | 0.15 | 1 | 0.02 | 0.19 |

| DSS, DPCA-PT | | | | | | | |
|---|---|---|---|---|---|---|---|
| | Inflam. Infil. | Gob. Cell Loss | Crypt Density Red. | Crypt Hyper-plasia | Muscle Thick. | Submuc. Inflam. | Total |
| | 2 | 3 | 3 | 0 | 0 | 1 | 8 |
| | 1 | 2 | 2 | 0 | 2 | 1 | 9 |
| | 1 | 2 | 2 | 1 | 1 | 1 | 9 |
| | 1 | 1 | 1 | 0 | 0 | 1 | 4 |
| | 1 | 0 | 0 | 0 | 0 | 1 | 3 |
| | 0 | 0 | 0 | 1 | 1 | 1 | 5 |
| | 1 | 1 | 0 | 1 | 1 | 1 | 6 |
| | 1 | 1 | 1 | 0 | 0 | 1 | 5 |
| | 1 | 1 | 1 | 1 | 1 | 1 | 7 |
| | 0 | 2 | 2 | 0 | 1 | 2 | 7 |
| | 3 | 2 | 2 | 1 | 1 | 2 | 7 |
| | 1 | 2 | 2 | 1 | 1 | 2 | 7 |
| Average | 1.08 | 1.42 | 1.33 | 0.5 | 0.75 | 1.25 | 6.42 |
| Stdev | 0.79 | 0.9 | 0.98 | 0.52 | 0.62 | 0.45 | 1.88 |
| p-value vs. DSS | 4.4E-3 | 4.8E-4 | 0.34 | 0.6 | 2.1E-4 | 6.0E-4 | 3.5E-3 |
| p-value vs. Healthy | 0.18 | 0.3 | 0.09 | 6.3E-4 | 0.78 | 1.5E-2 | 0.06 |

**Table S11:** IVIS normalized luminescence (p/s/sr) at Day 9 for Figure 3A.

| DSS | DSS, DPCA-RT | DSS, DPCA-PT |
|---|---|---|
| 11600 | 13600 | 32700 |
| 1770 | 107000 | 1130 |
| 168000 | 187000 | 0 |
| 7870 | 0 | 0 |
| 34300 | 30800 | 0 |

**Table S12:** ΔΔCt values for RT-qPCR of *MUC2* mRNA expression in Figure 3B.

| | Healthy | DSS | DSS, DPCA-RT | DSS, DPCA-PT |
|---|---|---|---|---|
| ΔΔCt | 1.43<br>0.92<br>0.76 | 1.29<br>1.25<br>0.85<br>1.35<br>1.54 | 1.50<br>1.52<br>2.00<br>2.27<br>1.43 | 1.54<br>1.83<br>1.77<br>1.44 |
| Average | 1.04 | 1.26 | 1.74 | 1.64 |
| Stdev | 0.35 | 0.25 | 0.37 | 0.19 |
| p-value vs. Healthy | -- | 0.333 | 0.038 | 0.029 |
| p-value vs. DSS | 0.333 | -- | 0.042 | 0.037 |

**Table S13:** IVIS normalized luminescence (p/s/sr) at Day 9 for Figure 4B.

| DSS | DSS, DPCA-RT |
|---|---|
| 106500 | 370000 |
| 156000 | 19430 |
| 376800 | 58270 |
| 55440 | 30450 |
| 149900 | 87470 |

**Table S14:** ΔΔCt values for RT-qPCR of *IL6* mRNA expression in Figure 4C.

| | Healthy | DSS | DSS, DPCA-RT |
|---|---|---|---|
| ΔΔCt | 0.61<br>0.91<br>1.82 | 7.21<br>9.65<br>9.38<br>4.23<br>4.56 | 4.47<br>2.89<br>1.25<br>2.48<br>1.03 |
| Average | 1.11 | 7.01 | 2.42 |
| Stdev | 0.63 | 2.57 | 1.39 |
| p-value vs. Healthy | -- | 0.009 | 0.18 |
| p-value vs. DSS | 0.009 | | 0.008 |

**Table S15:** ΔΔCt values for RT-qPCR of *TNFA* mRNA expression in Figure 4C.

| | **Healthy** | **DSS** | **DSS, DPCA-RT** |
|---|---|---|---|
| ΔΔCt | 1.16<br>1.08<br>0.76 | 6.02<br>2.89<br>3.43<br>2.35<br>5.78 | 2.93<br>1.44<br>1.37<br>1.01<br>0.88 |
| Average | 1 | 4.09 | 1.53 |
| Stdev | 0.21 | 1.69 | 0.82 |
| p-value vs. Healthy | -- | 0.022 | 0.33 |
| p-value vs. DSS | 0.022 | | 0.016 |

**Table S16:** ΔΔCt values for RT-qPCR of *IL10* mRNA expression in Figure 4E. Expression from one sample from DSS, DPCA-RT was 'undetermined' and excluded from analysis.

| | **DSS** | **DSS, DPCA-RT** |
|---|---|---|
| ΔΔCt | 1.15<br>0.83<br>0.94<br>2.88<br>0.39 | 6.35<br>5.05<br>7.24<br>2.02<br>n/a |
| Average | 1.24 | 5.16 |
| Stdev | 0.95 | 2.28 |
| p-value vs. DSS | -- | 0.0096 |

**Table S17:** ΔΔCt values for RT-qPCR of *CXCR4* mRNA expression in Figure 4E.

| | **DSS** | **DSS, DPCA-RT** |
|---|---|---|
| ΔΔCt | 1.13<br>0.89<br>0.97<br>1.70<br>0.61 | 2.39<br>1.77<br>1.64<br>1.49<br>1.19 |
| Average | 1.06 | 1.69 |
| Stdev | 0.41 | 0.45 |
| p-value vs. DSS | -- | 0.046 |

**Table S18:** ΔΔCt values for RT-qPCR of *CXCL12* mRNA expression in Figure 4E.

| | **DSS** | **DSS, DPCA-RT** |
|---|---|---|
| ΔΔCt | 1.24<br>0.81<br>0.85<br>1.10<br>1.07 | 0.85<br>0.67<br>0.97<br>0.93<br>1.34 |
| Average | 1.01 | 0.95 |
| Stdev | 0.18 | 0.25 |
| p-value vs. DSS | -- | 0.66 |

**Table S19:** ΔΔCt values for RT-qPCR of *EMT* factors mRNA expression in Figure 5B.

| | **Growth Media** | | | | | | **DPCA** | | | | | |
|---|---|---|---|---|---|---|---|---|---|---|---|---|
| | VIM | CDH1 | SNAI2 | TWIST1 | SNAI1 | TGFB | VIM | CDH1 | SNAI2 | TWIST1 | SNAI1 | TGFB |
| | 0.97<br>1.03 | 0.83<br>1.20 | 0.87<br>0.98<br>1.17 | 1.04<br>0.88<br>1.09 | 1.11<br>0.74<br>1.22 | 1.01<br>0.94<br>1.05 | 2.86<br>2.17<br>2.49 | 0.13<br>0.12<br>0.09 | 9.43<br>4.55<br>7.60 | 3.35<br>1.65<br>2.37 | 2.13<br>1.42<br>0.45 | 2.73<br>1.20<br>2.46 |
| Avg | 1.00 | 1.02 | 1.01 | 1.00 | 1.02 | 1.00 | 2.50 | 0.11 | 7.19 | 2.46 | 1.33 | 2.13 |
| Stdv | 0.04 | 0.26 | 0.15 | 0.11 | 0.25 | 0.06 | 0.35 | 0.02 | 2.46 | 0.85 | 0.84 | 0.81 |
| p-val | -- | -- | -- | -- | -- | -- | 1.0E-2 | 7.3E-3 | 1.2E-2 | 4.3E-2 | 0.57 | 0.075 |

**Table: S20:** Western blot quantification in Figure 6B.

| | **Growth Media** | **DPCA** |
|---|---|---|
| | 0.46 | 0.56 |
| | 0.49 | 0.58 |
| | 0.47 | 0.61 |
| Avg | 0.47 | 0.58 |
| Stdev | 0.012 | 0.023 |
| p-val | | 0.0019 |

**Table S21-22:** ΔΔCt values for RT-qPCR for DPCA target mRNA expression in Figure 6C.

| **Growth Media** | | | | | | | | | |
|---|---|---|---|---|---|---|---|---|---|
| | TFF3 | PGP | CLDN1 | CD73 | ITGA2 | ITGA6 | ITGB1 | MMP9 | TIMP1 |
| | 1.05<br>0.95 | 1.12<br>0.90 | 1.14<br>1.07<br>0.82 | 1.05<br>0.95 | 0.72<br>1.81<br>0.76 | 0.82<br>1.50<br>0.81 | 0.99<br>0.81<br>1.23 | 0.48<br>1.63<br>1.29 | 0.95<br>0.99<br>1.06 |
| Avg | 1.00 | 1.01 | 1.01 | 1.00 | 1.09 | 1.04 | 1.02 | 1.13 | 1.00 |
| Stdev | 0.074 | 0.018 | 0.17 | 0.069 | 0.61 | 0.39 | 0.21 | 0.59 | 0.057 |

| **DPCA** | | | | | | | | | |
|---|---|---|---|---|---|---|---|---|---|
| | TFF3 | PGP | CLDN<br>1 | CD73 | ITGA2 | ITGA6 | ITGB1 | MMP9 | TIMP1 |
| | 3.39<br>2.29<br>2.18 | 1.90<br>1.72<br>2.29 | 1.82<br>1.63<br>1.99 | 3.07<br>2.88<br>4.00 | 11.6<br>12.8 | 30.7<br>21.4 | 4.11<br>3.78 | 3.91<br>7.14 | 3.05<br>2.66 |
| Avg | 2.62 | 1.97 | 1.81 | 3.32 | 12.20 | 26.05 | 3.95 | 5.53 | 2.85 |
| Stdev | 0.67 | 0.3 | 0.18 | 0.60 | 0.90 | 6.58 | 0.23 | 2.29 | 0.28 |
| p-val | 0.049 | 0.026 | 4.9E-3 | 0.014 | 0.005 | 0.037 | 0.004 | 4.2E-2 | 1.2E-3 |

**Table S23:** FITC-dextran concentration for Caco-2 permeability assay in Figure 6C.

| | **Growth Media** | | **DPCA** | |
|---|---|---|---|---|
| Time (hours) | FITC-Dextran Conc. (µg/mL) | Stdev | FITC-Dextran Conc. (µg/mL) | Stdev |
| 0.50 | 0.61 | 0.04 | 0.56 | 0.08 |
| 1.00 | 0.96 | 0.11 | 0.76 | 0.03 |
| 1.50 | 1.18 | 0.21 | 0.87 | 0.04 |
| 2.00 | 1.34 | 0.17 | 0.98 | 0.03 |
| 2.50 | 1.45 | 0.11 | 1.05 | 0.04 |
| 0.50 | 0.61 | 0.04 | 0.56 | 0.08 |

## SI References


1. Cheng, J., Amin, D., Latona, J., Heber-Katz, E. & Messersmith, P. B. Supramolecular Polymer Hydrogels for Drug-Induced Tissue Regeneration. *ACS Nano* **13**, 5493–5501 (2019).
